# *Bifidobacterium longum* subsp. *infantis* utilizes human milk urea to recycle nitrogen within the infant gut microbiome

**DOI:** 10.1080/19490976.2023.2192546

**Published:** 2023-03-26

**Authors:** Xiaomeng You, Asha Rani, Ezgi Özcan, Yang Lyu, David A. Sela

**Affiliations:** a Department of Food Science, University of Massachusetts, Amherst, MA, USA; b Department of Microbiology, University of Massachusetts, Amherst, MA, USA; c Department of Nutrition, University of Massachusetts, Amherst, MA, USA; d Department of Microbiology and Physiological Systems, University of Massachusetts Chan Medical School, Worcester, MA, USA

**Keywords:** Microbiota, human milk, urea, bifidobacteria, nitrogen metabolism

## Abstract

Human milk guides the structure and function of microbial commensal communities that colonize the nursing infant gut. Indigestible molecules dissolved in human milk establish a microbiome often dominated by bifidobacteria capable of utilizing these substrates. Interestingly, urea accounts for ~15% of total human milk nitrogen, representing a potential reservoir for microbiota that may be salvaged for critical metabolic operations during lactation and neonatal development. Accordingly, *B. infantis* strains are competent for urea nitrogen utilization, constituting a previously hypothetical phenotype in commensal bacteria hosted by humans. Urease gene expression, downstream nitrogen metabolic pathways, and enzymatic activity are induced during urea utilization to yield elevated ammonia concentrations. Moreover, biosynthetic networks relevant to infant nutrition and development are transcriptionally responsive to urea utilization including branched chain and other essential amino acids. Importantly, isotopically labeled urea nitrogen is broadly distributed throughout the expressed *B. infantis* proteome. This incisively demonstrates that the previously inaccessible urea nitrogen is incorporated into microbial products available for infant host utilization. In aggregate, *B. infantis* possesses the requisite phenotypic foundation to participate in human milk urea nitrogen recycling within its infant host and thus may be a key contributor to nitrogen homeostasis early in life.

## Introduction

Commensal microbiota colonize the postnatal infant gastrointestinal tract (GIT) to participate in gut tissue maturation, interconnected metabolic operations, immunological development, and other functions that contribute to host homeostasis. ^1^ In addition to direct nourishment of the infant, exogenous molecules dissolved in milk interact with autochthonous microbial populations along the GIT to modulate host physiology. Accordingly, infant-indigestible human milk glycans,^2^ and potentially other solutes such as peptides,^3^ dictate the form and function of the infant gut microbiome. In turn, compounds derived from microbial metabolism (e.g. vitamins, organic acids, antimicrobials, etc.) provide resources during the rapid growth experienced during early infant development.^4^

Specific bifidobacterial lineages have evolved to colonize the nursing infant GIT and thus are often abundant within this environment.^5^ Microbiologically-active molecules secreted in human milk enhance bifidobacterial fitness relative to competing genera.^2,6^ These milk-microbial-infant interactions are exemplified by the human milk oligosaccharides (HMOs). Rather than contributing directly to infant nutrition, HMOs transit intact to the colon and are metabolized by select bifidobacterial populations to accumulate biomass and generate ATP.^6,7^ *Bifidobacterium longum* subsp. *infantis* (*B. infantis*) efficiently metabolizes HMOs as a sole carbon source which is currently understood to be an exceedingly rare population-level phenotype. The innovation of this nutritive strategy is likely the consequence of co-evolution between *B. infantis*, their infant host, and human milk provided by the mother.^6,8^

Carbohydrates localized to the GIT are acquired exogenously from diet, decorate apical epithelial surfaces, and are secreted in glycoconjugate macromolecules (e.g. mucins).^9^ To date, carbon metabolism within the microbiome has received the greatest scientific scrutiny in characterizing diet-host-microbial systems.^10^ This focus has considerably resolved the role of fermentative microbes within their ecological niche. As a result, bifidobacterial physiology is almost fully understood as a function of carbohydrate metabolism.^11^ Nitrogen metabolism within the microbiome, in contrast, remains relatively underexplored in integrating infant nutrition with commensal biology and attendant ecological interactions.

Protein incorporates the primary dietary nitrogen delivered in human milk (0.9–1.2 g/100 ml).^12^ The developing infant digestive system may have lower pancreatic proteases secreted relative to adults.^13^ Thus, a fraction of these proteins, notably lactoferrin and secretory IgA, remain stable while transiting the infant GIT to exert a local bioactive function.^14^ The remaining complement of protein nourishes the infant through satisfying nitrogen and amino acid demands. Maintaining nitrogen homeostasis could be challenged, however, when milk protein concentrations diminish over the course of lactation^15^ or due to increased demands of preterm birth.

A diverse assortment of non-protein nitrogen (NPN) is incorporated into human milk accounting for ~25% of total nitrogen, with urea comprising ~50% of the NPN fraction.^16^ Moreover, urea is secreted into the colonic lumen as a consequence of infant basal metabolism.^17^ It is estimated that infants excrete ~20% of urea in urine whereas a much larger fraction is retained within the colon and thus available for bacterial hydrolysis.^18^ Invariably, *B. infantis* genomes encode an urease gene cluster and transporters predicted to translocate urea and its nickel cofactor.^6,8^ Similar loci linked to urease function are relatively rare in commensal bacteria that typically colonize the infant gut microbiome and could potentially catalyze intracellular urea hydrolysis. Accordingly, we hypothesize that ureolytic *B. infantis* liberate ammonia from sequestered urea to be used as an anabolic substrate in protein biosynthesis. Therefore, urea would be reclaimed through a bifidobacterial-mediated process referred to as urea nitrogen recycling or salvaging. This heretofore understudied metabolic process in humans would enable the infant to harness an otherwise inaccessible nitrogen pool.

To test this hypothesis, the extent to which *B. infantis* utilizes urea nitrogen was systematically characterized. Evidence in support of bifidobacterial-mediated urea nitrogen recycling includes a comprehensive description of the *B. infantis* urea utilization phenotype. In addition to *in vitro* growth dynamics, systems-level approaches yielded urea-specific activation of global transcriptional programs, impacts to central fermentative processes, incorporation of urea nitrogen into the proteome, differential metabolomics linked to nitrogen source, and an initial characterization of host-microbial interactions.

## Results

### The infant gut commensal *Bifidobacterium longum* subsp. *infantis* utilizes urea as a primary nitrogen source

**Figure 1.**
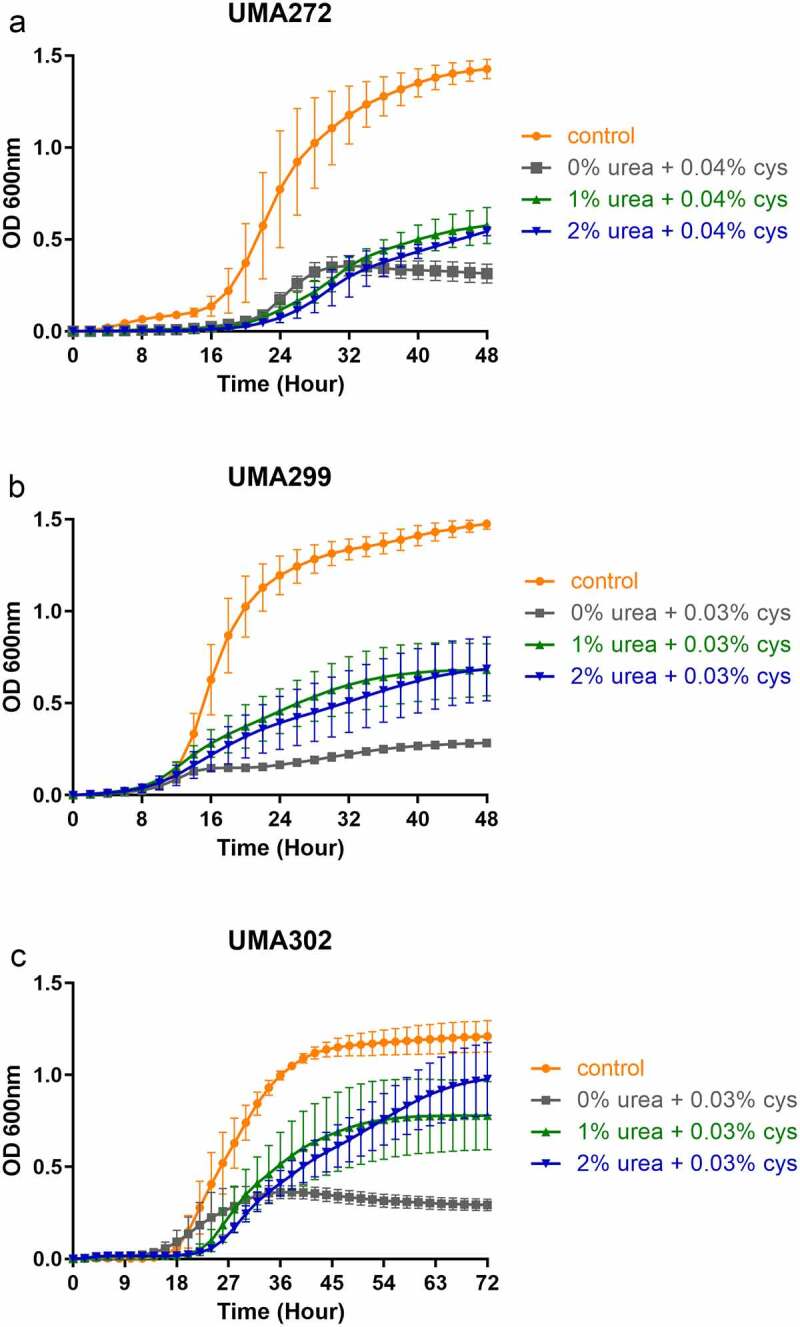
***B. infantis* biomass accumulation while utilizing urea as a nitrogen source**. UMA272 **(a)**, UMA299 **(b)**, and UMA302 **(c)** growth on urea as the primary nitrogen source. Three biological replicates were evaluated with results presented as mean ± SD. cys, L-cysteine.

*B. infantis* strains were evaluated for urea utilization as a primary nitrogen source. As *B. infantis* is auxotrophic for L-cysteine, this amino acid was added in limiting concentrations to restrict its use as a general nitrogen source. Significantly, all *B. infantis* strains utilized urea beyond the limiting L-cys to demonstrate urea is utilized as a primary nitrogen source (Figure 1 and Table S1). Specifically, higher cellular biomass was achieved relative to the urea-deficient medium for 1.0% urea (asymptotic OD_600 nm_ = 0.57 ± 0.11–0.77 ± 0.20) and 2.0% urea (OD_600 nm_ = 0.54 ± 0.07–0.98 ± 0.19) (*p* < 0.05) (Table S1). Among the *B. infantis* strains tested, UMA302 grew most vigorously with an asymptotic OD_600 nm_ = 0.77 ± 0.20 (1.0% urea) and 0.98 ± 0.19 (2.0% urea). Interestingly, the growth rate on urea did not significantly diverge from that of the complex nitrogen control for *B. infantis* UMA272. This suggests that *B. infantis* does not exhibit a relative preference for the complex nitrogen source, which is somewhat unexpected given the greater biosynthetic investment necessitated to utilize urea. In contrast, the *B. infantis* UMA299 growth rate on urea was significantly lower relative to the positive control (*p* < 0.05). This is consistent with the divergence of UMA299 from other *B. infantis* isolates with regard to milk solute utilization, as this strain does not efficiently metabolize HMO.^6,19^ Nevertheless, all *B. infantis* strains tested utilize urea as a primary nitrogen source to provide the foundational support for bifidobacterial-mediated urea nitrogen recycling (UNR) within the infant gut microbiome.

### Urea utilization induces elevated urease activity and ammonia accumulation

**Figure 2.**
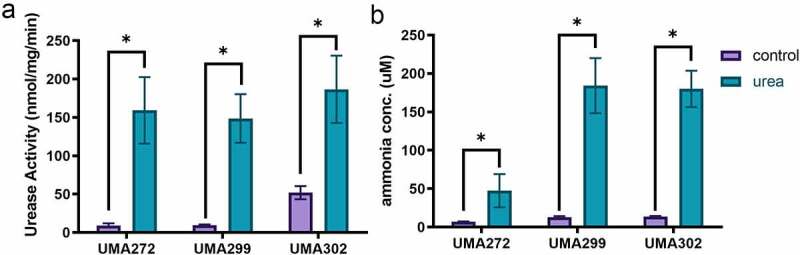
**Urease activity (a) and ammonia production (b) while subsisting on urea as a nitrogen source**. at least three biological replicates were assayed with results presented as mean ± SD (Welch’s t-test; *, *p* < 0.05).

As *B. infantis* utilizes urea as a primary nitrogen source, it is likely that the rate-limiting metabolic step is catalyzed via urease hydrolysis (EC 3.5.1.5) to yield carbon dioxide and ammonia. Accordingly, comparative genomics identified a co-linear urease gene cluster that is conserved in all *B. infantis* strains analyzed to date.^6,8^ In order to assess urease function during urea metabolism, ureolytic activity was measured in cell-free lysates harvested during mid-exponential growth (Figure 2A). Urease activity is significantly induced by urea relative to the complex nitrogen control in UMA272 (17.20-fold), UMA299 (15.30-fold), and UMA302 (3.59-fold) (*p* < 0.05). UMA302 exhibited higher constitutive urease activity under control conditions to explain the relatively modest, albeit significant, induction. Clearly, *B. infantis* urease activity is induced by its substrate during urea utilization.

Ammonia concentrations at stationary phase indicates utilization efficiency as a function of urease-catalyzed urea metabolism (Figure 2B). Consistent with urease activity, significantly higher ammonia production was observed in all *B. infantis* strains (*p* < 0.05) and induced between 6.80–14.30-fold in a strain-dependent manner. Interestingly, UMA272 exhibited the lowest induction of ammonia production (6.80-fold) whereas its urease activity was induced to the greatest magnitude (17.20-fold). It is possible that UMA272 efficiently assimilated a greater fraction of ammonia for nitrogen anabolism relative to the other strains.

### Urea nitrogen utilization is interconnected with carbon metabolism

**Figure 3.**
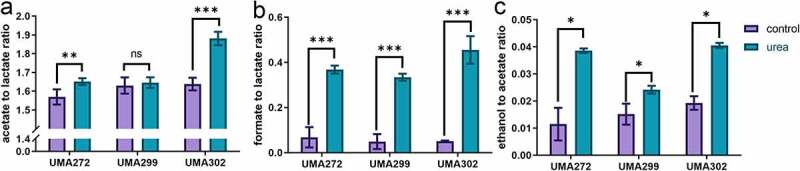
**Fermentative endproducts secreted during urea nitrogen utilization**. Carbohydrate metabolism endproducts were quantified by HPLC and normalized as ratios of acetate to lactate **(a)**, formate to lactate **(b)**, and ethanol to acetate **(c)**. Four biological replicates were evaluated with results presented as mean ± SD. Two-way ANOVA was performed for (A & B) and Welch’s t-test was performed for (C). *, *p* < 0.05, ***, *p* < 0.001.

Bifidobacteria ferment carbohydrates through a characteristic fermentative pathway that terminates in secretion of organic acids and other endproducts that interact with infant and heterologous microbiota. Acetate and lactate are secreted in a theoretical ratio of 1.5, with ethanol and formate produced to a lesser extent, and typically during inefficient carbohydrate metabolism.^11^ Thus we postulated that if bifidobacteria nitrogen and carbon metabolism is linked, urea utilization would promote formate production to accommodate downstream biosynthetic investments in harnessing ammonia nitrogen. To test this hypothesis, fermentative endproducts were quantified subsequent to urea nitrogen utilization (Figure 3 and Fig. S1).

As expected, *B. infantis* approaches the acetate:lactate theoretical ratio of 1.5 under control nitrogen conditions. Interestingly, UMA272 and UMA302 significantly increased (*p* < 0.05) their secreted acetate:lactate ratio during urea utilization  , from 1.57 ± 0.04 to 1.65 ± 0.02 and from 1.64 ± 0.03 to 1.88 ± 0.04, respectively (Figure 3A). We previously reported similar metabolic shifts during carbohydrate metabolism to address an energetic deficit by shunting pyruvate toward acetyl-CoA at the expense of lactate.^11,20^ In contrast, UMA299 did not exhibit a similar physiological reorientation. Again, this is consistent with other variant metabolic phenotypes while utilizing human milk molecules.

In general, formate secretion is negatively correlated with lactate production, and it increases NAD+ recycling during inefficient carbohydrate metabolism.^20,21^ During urea nitrogen metabolism, all *B. infantis* strains produced significantly higher formate:lactate ratios regardless of final biomass achieved (*p* < 0.05) (Figure 3B). The ratio of ethanol:acetate ratio also increased independent of strain (*p* < 0.05) (Figure 3C). That urea metabolism prompts a shift in the carbohydrate metabolite profile demonstrates that carbon and nitrogen metabolism are linked in bifidobacteria. More specifically, urea nitrogen and central fermentative metabolism are interconnected potentially to satisfy ATP demands during *de novo* biosynthesis of nitrogenous precursors from ammonia.

### Urea nitrogen is assimilated into the *Bifidobacterium longum* subsp. *infantis* proteome

**Figure 4.**
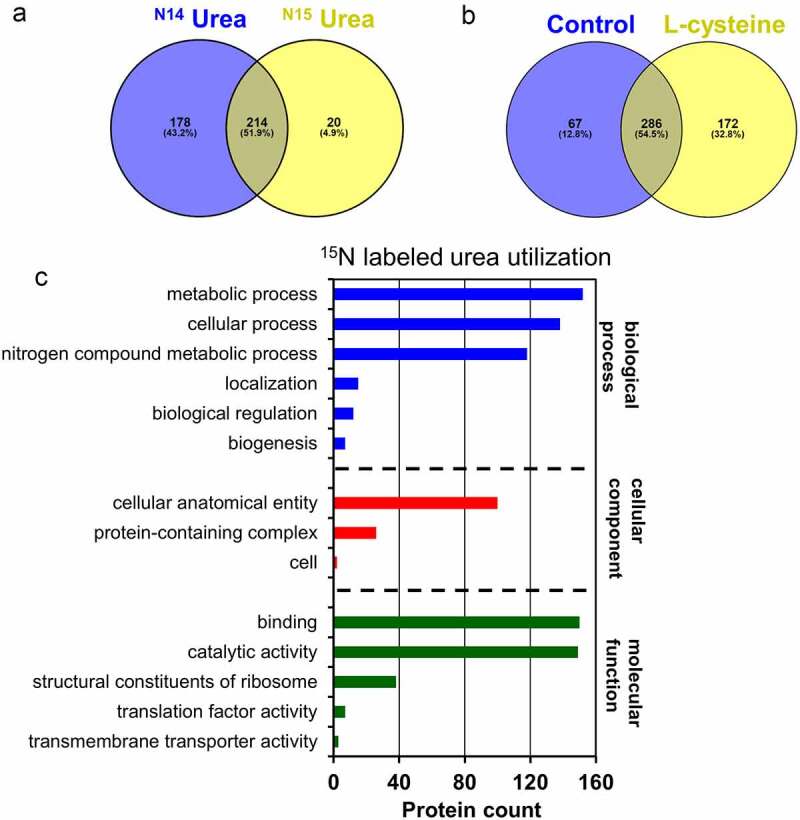
**^15^N-labeled proteins identified while subsisting on isotopically labeled urea**. Venn diagram of proteins shared between labeled and unlabeled urea proteomes **(a)**, proteins shared between the complex nitrogen control and L-cysteine proteomes **(b)**, and urea nitrogen incorporated into ^15^N-labeled proteins assigned to a gene ontology category **(c)**.

Mass spectrometry was used to determine the metabolic fate of ^15^N-labeled urea during intracellular utilization as a primary nitrogen source (Fig. S2). A total of 234 proteins incorporate the ^15^N-label in the expressed *B. infantis* proteome during utilization of ^15^N-labeled urea (Table S2-S3). A total of 392 proteins were identified while subsisting on unlabeled urea, with 214 proteins (51.9%) common to both datasets (Figure 4A). Thus it is clear that urea nitrogen is reclaimed and enters biosynthetic pathways, and is incorporated into the *B. infantis* proteome as a potential terminus.

^15^N-labeled urea nitrogen enters a wide range of processes conventionally classified as cellular, metabolic, and biosynthetic and includes housekeeping processes such as ribosomal subunits. This involves amino acid biosynthesis, amino-sugar metabolism, carbohydrate metabolism, cofactor biosynthesis, glycan biosynthesis, metabolic intermediate biosynthesis, one carbon metabolism, and purine and pyrimidine metabolism (Table S4). Of note, urea nitrogen enters cellular operations assigned a function in maintaining nitrogen homeostasis (Fig. S3 and 4C).

*B. infantis* grown on L-cysteine serves as a reference proteome in comparison to urea. As *B. infantis* is auxotrophic for L-cysteine, it is necessary to add this limiting amino acid to all growth conditions with differentially expressed proteins reported in Table S5. A total of 29 proteins were upregulated (log2 fold change >1, FDR <0.05) during urea utilization relative to L-cysteine alone. Proteins upregulated during urea utilization include glutamate synthase (encoded by gene Blon_1481; log2FC = 5.7, FDR <0.05) and several ABC transporter-associated extracellular solute-binding proteins, among others. Glutamate synthase (EC 1.4.1.14) is highly significant as it catalyzes ammonium assimilation within the GS/GOGAT cyclical pathway that synthesizes glutamate and glutamine to serve as nitrogen donors to satisfy biosynthetic requirements. Moreover, 2-oxoglutarate is a nitrogen carrier and provides a carbon backbone in the GS/GOGAT system to potentially link urea nitrogen and carbon metabolism. The *B. infantis* genome encodes a partial tricarboxylic acid cycle with the requisite genes to synthesize 2-oxoglutarate, despite not performing respiration. In summation, urea nitrogen is broadly distributed throughout the *B. infantis* proteome, likely through an ammonium intermediate via the GS/GOGAT system.

Specific proteins that integrate ^15^N-labeled urea are within pathways related to nitrogen metabolism or are otherwise of interest. This includes L-lysine biosynthesis (4-hydroxy-tetrahydrodipicolinate synthase(Blon_2034)), glutamate biosynthesis (Blon_1482), carbohydrate degradation (ribose-5-phosphate isomerase A (Blon_2191), phosphoglycerate kinase (Blon_1087), and 2,3-bisphosphoglycerate-dependent phosphoglycerate mutase (Blon_2152)). Cofactor biosynthesis (Pyridoxal 5’-phosphate synthase(Blon_1994)) and protein biosynthesis (Elongation factor P(Blon_0735)) were identified as well (Table S3). A detailed list of ^15^N-labeled proteins identified in metabolic pathways is provided in Table S3-S4.

^15^N-labeled L-cysteine served as the sole nitrogen source with labeled nitrogen incorporated into the *B. infantis* proteome (Fig. S4A, Fig. S5). This analysis identified ~24% (*n* = 92) of the^15^N-labeled proteins in the *B. infantis* proteome while utilizing^15^N-labeled L-cysteine alone (Fig. S4A, and S5). This suggests that minimal amount of L-cysteine added to the media is incorporated into the *B. infantis* proteome (~36.3%, 87), however to a lesser extent compared to^15^N-labeled urea nitrogen (~97.6%, *n* = 148) (FigureS4B, Tables S7 and S8).

*B. infantis* grown on complex nitrogen with L-cysteine was compared to L-cysteine alone to characterize the proteome while growing on a rich medium (i.e. control). This yielded ~55% (*n* = 286) proteins shared between the rich medium and L-cysteine alone. Interestingly, the cells grown in the rich medium exhibited 12% (*n* = 67) of unique proteins whereas L-cysteine alone provided significantly more at 33% (*n* = 172) (Figure 4B). This indicates that a greater fraction of the proteome’s total potential is mobilized while *B. infantis* is metabolizing L-cysteine. This sole nitrogen source necessitates a broader anabolic program as per the need to biosynthesize a greater array of nitrogenous products (e.g. amino acids).

In the cells provided complex nitrogen, there is greater representation of fundamental processes within the proteome including cell division, translation, nucleic acid binding, integral components of membrane, ribosomal among various uncharacterized proteins (Table S6). This suggests that cellular requirements are being fulfilled to the extent by which cell growth and division is promoted. This contrasts with the L-cysteine proteome with its upregulated proteins involved in nitrogen acquisition and metabolism which is consistent with a shift in cellular physiological priorities. This includes ABC transporters, urease components (e.g. alpha subunit), nitrogen regulatory protein P-II, extracellular solute-binding proteins, an ammonium transporter among uncharacterized proteins (Table S6). It is noteworthy that glutamate synthase (Blon_1481) was significantly upregulated (log2FC = 5.7, FDR <0.05, Table S5) only during urea utilization, to promote ammonium assimilation while utilizing urea as a sole nitrogen source.

### Urea nitrogen metabolism induces a unique transcriptional program

**Figure 5.**
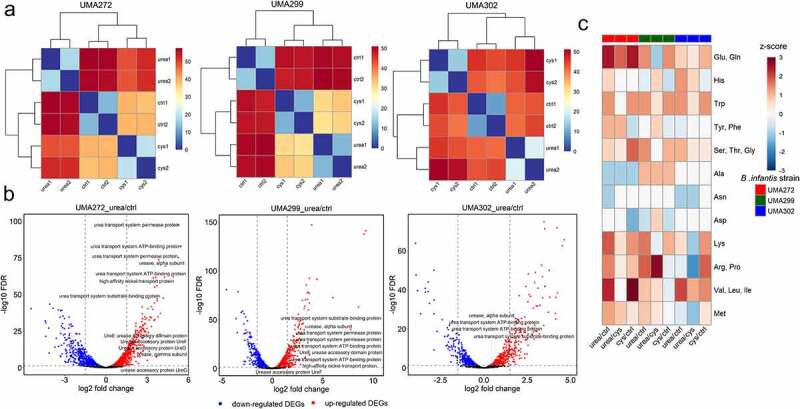
**The global transcriptome response during urea nitrogen utilization**. Euclidian distances between *B. infantis* transcriptomes during urea nitrogen utilization **(a)**, differentially expressed genes between urea relative to control nitrogen **(b)**, and amino acid biosynthetic pathways regulated during urea utilization (z-scores). cys, L-cysteine, ctrl, control (complex nitrogen). 1, first biological replicate, 2, second biological replicate.

*B. infantis* transcriptomes were profiled through RNAseq to identify physiological processes contributing to, and responding to, urea utilization. The average raw read depth ranged from 10.81 to 18.50 million, to achieve the threshold to interrogate transcriptomes at meaningful resolution.^22^ Reads were aligned to the reference ATCC 15697^T^ genome, resulting in alignment rates of 97.34% for UMA272, 89.13% for UMA299, and 85.67% for UMA302 (Table S9). The transcriptomes were evaluated by hierarchical clustering (Figure 5A) and principal component analysis (PCA) (Fig. S6). Growth on urea was compared to complex nitrogen, as well as L-cysteine as the sole nitrogen source. The latter served as an additional defined nitrogen source, and moreover, discerns features to urea metabolism as L-cysteine was present in all conditions.

As expected, biological replicates clustered, and the transcriptomes exhibit distinct expression profiles dependent on nitrogen source. In aggregate analysis, strain identity drove clustering (Fig. S7) regardless of nitrogen source and transcriptomes segregated unambiguously according to isolate (Fig. S8). Differentially expressed genes (DEGs) were identified and cataloged in Table S10. *B. infantis* UMA272 possesses the highest density of DEGs while metabolizing urea relative to the complex nitrogen control (1376, FDR <0.05), followed by UMA299 (1064, FDR <0.05) and UMA302 (876, FDR <0.05). UMA299 exhibited considerably less DEGs in the comparison between urea and L-cysteine than the other strains (FDR < 0.05) (Table S10). The similarity between defined nitrogen conditions may indicate constitutive processes are fundamental in the strain’s physiological response. This suggests that UMA299 deploys a general transcriptional response to distribute limited nitrogen across integrated biosynthetic networks. This is illustrative for UMA272 as DEGs between L-cysteine and the complex control yielded only 774 DEGs (FDR <0.05), significantly lower than DEGs between urea relative to the control. This denotes that DEGs responded to the urea molecule and its physicochemical form, rather than a general response as was observed with UMA299.

### Urea induces urease gene cluster expression for substrate acquisition and hydrolysis

The *B. infantis* chromosome encodes a 12 gene locus including genes predicted for urea transport, nickel transport, urease accessory genes, and urease structural and catalytic genes (Figure 5B, Fig. S9 and Table S11).^6,8^ These genetic determinants are likely essential in acquisition and metabolism of extracellular urea and are depicted in (Fig. S10).

Urea significantly upregulated putative urea transport genes (Blon_0104 – Blon_0108) in UMA272 and UMA302. This is in comparison to both L-cysteine (average log2FC = 2.98) and complex nitrogen (average log2FC = 5.18) for UMA272 (FDR < 0.05). Urea prompts UM302 to upregulate all transport genes relative to L-cysteine, and 3 of the 5 genes were induced compared to complex nitrogen. Although UMA299 significantly upregulated urea transport genes relative to the control, they are downregulated relative to when metabolizing L-cysteine (average logFC = −1.30, FDR <0.05). It is possible that UMA299 prefers L-cysteine as a nitrogen substrate, or that this modest downregulation is artifactual. An alternative explanation is that nitrogen starvation experienced in L-cysteine metabolism may induce UMA299 to urea acquisition and potentially other nitrogenous molecules from its environment. A similar pattern emerged for a putative high-affinity nickel transport gene (Blon_0109), which likely translocate this essential urease metal cofactor. Accordingly, urea induces *B. infantis* UMA272 and UMA302 to upregulate Blon_0109 relative to L-cysteine. Whereas UMA299 downregulates the locus in the same comparison.

Accessory proteins assemble the nickel metallocenter within the urease active site. Accordingly, UMA272 upregulates Blon_0112-Blon_0115 during urea metabolism relative to L-cys (average log2FC = 1.62, FDR <0.05) and complex nitrogen (average log2FC = 2.20, FDR <0.05). Interestingly, *B. infantis* UMA 302 did not alter expression of these accessory genes, regardless of comparison conditions, with the exception of Blon_0112 which was downregulated relative to L-cys (logFC = −0.66, FDR <0.05). This indicates that these genes are likely constitutively expressed at a level sufficient to enable urease function. As with other urea-related genes, all four accessory genes were downregulated within the UMA299 transcriptome relative to L-cys alone (average logFC = −1.01, FDR <0.05).

The active urease enzyme consists of three subunits encoded by Blon_0110 (β and γ subunits) and Blon_0111 (α subunit). Urea significantly induces upregulation of both genes in UMA272 regardless of the comparison conditions (log2FC ≥ 2.78, FDR <0.05). Similarly, UMA302 upregulated Blon_0111 during urea metabolism, although Blon_0110 expression did not vary. UMA299 exhibited a mixed expression profile that was only moderately upregulated or downregulated depending on the conditions.

### Urea metabolism upregulates ammonia assimilation pathways

Urea hydrolysis yields ammonia to feed into biosynthetic pathways requiring organic nitrogen. Accordingly, *B. infantis* is predicted to assimilate ammonia via a two-component regulatory system involving the proteins PII-uridylyltransferase (Blon_0225, *glnD*) and nitrogen regulatory protein N-II (Blon_0224, *glnB*).^23^ *B. infantis* UMA272 upregulates both genes during urea metabolism regardless of comparative conditions (log2FC = 1.39–3.72, FDR <0.05). UMA 302 exhibits a mixed profile in that both genes are downregulated relative to L-cys whereas Blon_0224 is upregulated relative to the complex nitrogen control (log2FC = 0.73, FDR <0.05). *B. infantis* UMA299 generally downregulates these genes during urea metabolism (logFC = −1.00 to −0.67, FDR <0.05).

A primary entry point for ammonia assimilation are the amino acids glutamate and glutamine which are foundational in the biosynthesis of other amino acids and serve as intracellular nitrogen donors. Thus, the degree to which their bioysynthetic pathway is upregulated provides insight into the metabolic fate of urea-derived nitrogen ammonia (Figure 5C. Table S12 and Table S14). Accordingly, all three strains upregulated the two pathways relative to the complex nitrogen control with z scores ranging from 0.71 to 2.41 depending on the strain. Moreover, L-cys as the sole nitrogen source upregulated the same biosynthetic pathways in all three strains relative to the complex control. This is consistent with urea inducing translation of the key enzyme glutamate synthase (Blon_1481) as identified in comparative proteomics. Interestingly, and again linked with variant UMA299 physiology, both UMA272 and UMA302 upregulated glutamate/glutamine biosynthesis during urea metabolism relative to L-cys whereas UMA299 did not exhibit the same response. Instead, UMA299 downregulated glutamate/glutamine biosynthesis (z score = −1.06) while utilizing urea relative to L-cysteine.

### Urea metabolism increases demand for amino acid biosynthesis including those essential for host nutrition

*B. infantis* synthesizes all amino acids from ammonium with the notable exception of L-cysteine. In total, 78 genes are predicted to be directly involved in amino acid biosynthesis (Fig. S11 Table S12 and Table S13). *De novo* synthesis potentially provides an additional pool of amino acids to other members of the microbiome, as well as the developing infant during a critical window of rapid development. It was therefore predicted that amino acid biosynthetic networks would be upregulated in response to a defined sole nitrogen source, be it urea or L-cysteine. Among the 78 amino acid biosynthetic genes, *B. infantis* UMA272 exhibits 52 DEGs during urea metabolism relative to the complex nitrogen control and 35 DEGs relative to L-cysteine (FDR < 0.05). Similarly, L-cysteine metabolism relative to the complex nitrogen yielded 43 DEGs (FDR < 0.05). The overwhelming majority of DEGs are upregulated regardless of defined nitrogen substrate (≥84.6%). Moreover, and generalizing the trend, the UMA302 and UMA299 transcriptomes contained considerable numbers of DEGs related to amino acid biosynthesis with most exhibiting upregulation while utilizing urea (FDR < 0.05) (Table S13 and Table S14).

Interestingly, urea metabolism upregulates branched-chain amino acids (BCAA) biosynthesis in all strains relative to the complex control (z score range = 0.87–2.31) (Figure 5C and Table S14). The BCAAs valine, leucine, and isoleucine promote insulin secretion and β-cell mass expansion required for mTORC1-driven postnatal growth.^24^ These amino acids are not synthesized by the infant host and is therefore essential to obtain these exogenously to satisfy metabolic requirements.

Tryptophan is a precursor of serotonin and thus critical for central nervous system development as it modulates infant behavior.^25^ Accordingly, biosynthetic pathways for this essential amino acid are identically upregulated by the three *B. infantis* strains subsisting on urea relative to the complex nitrogen control (z = 1.34). Interestingly, L-cysteine induced the same upregulation pattern relative to the control conditions (z = 1.34). Furthermore, lysine biosynthesis was upregulated in all *B. infantis* strains while utilizing urea (z = 0.53–1.87) relative to the complex control with a similar pattern observed with L-cysteine.

Consistent with the transcriptome data, the labeled proteome included proteins involved in L-lysine biosynthesis such as 4-hydroxy-tetrahydrodipicolinate reductase, further confirming that urea nitrogen is incorporated into lysine biosynthesis in *B. infantis* (Table S3). Similar to tryptophan, lysine is an essential amino acid and was previously demonstrated that ^15^Nfrom isotopically-labeled urea is integrated into lysine and subsequently transferred to the infant.^26,27^ This is consistent with the evidence presented herein for bifidobacterial-mediated UNR.

### Urea metabolism elicits strain-level variation in amino acid biosynthesis

Urea induces all *B. infantis* strains to synthesize BCAAs, and nitrogen limiting conditions (i.e. urea or L-cysteine) induce trypothan and lysine biosynthetic pathways. Strain-dependent variation, however, was observed for most amino acids during urea metabolism compared to L-cysteine (Figure 5C). *B. infantis* UMA272 and *B. infantis* UMA299 exhibit similar trends for several amino acid pathways. Accordingly, tyrosine, phenylalanine, arginine, proline, and methionine biosynthesis pathways are upregulated by urea (z = 0.3–2.71). However, the overlapping pathway for arginine/proline and methionine biosynthesis are downregulated in *B. infantis* UMA302 (z = −1.81 andz = −1.22, respectively). Urea metabolism leads to upregulation in serine, glycine, and threonine biosynthesis in UMA272 (z = 0.35) and UMA302 (z = 0.35), with these pathways remaining constant in UMA299. The asparagine biosynthesis pathway remains unchanged for UMA272 and UMA299, with UMA302 experiencing downregulation during urea metabolism (z = −1.00). UMA299 upregulates aspartate biosynthesis in response to urea (z = 0.71), whereas UMA272 and UMA302 do not.

### Urea nitrogen utilization modulates carbohydrate transcriptional programs

Urea metabolism increases carbon flux through secretion of acetate, formate, and ethanol at the expense of lactate, potentially regulated at gene transcription. Bifidobacterial central fermentative pathway involves enzymes encoded by 16 putative genes (Fig. S12).^8^ During urea utilization, UMA272 and UMA302 exhibit 8 and 10 DEGs of the 16 fermentative genes respectively compared with complex nitrogen, which are all upregulated during urea utilization. UMA299, in contrast, upregulates 8/14 DEGs under the same conditions, with the remaining 6 DEGs downregulated.

The majority of DEGs identified in comparing urea against L-cysteine growth represent upregulated genes (UMA 272, 5/6; UMA302, 3/3; UMA299, 10/10). This suggests a physiological imperative to increase carbohydrate metabolism during urea utilization beyond that of a defined nitrogen source. Blon_1731 encodes an acetate kinase that converts acetyl-CoA to acetate and is upregulated in the all strains during urea utilization relative to the complex nitrogen (log2FC range = 0.80–1.21, FDR <0.05). This is consistent with the shift toward greater acetate/lactate production ratio (Figure 3A).

Urea promotes greater formate secretion (Figure 3B and Fig. S1C) and both UMA272 and UMA299 upregulated a formate acetyltransferase gene (Blon_1715) regardless of the transcriptome comparison. In contrast, UMA302 did not exhibit the same expression modulation. Greater ethanol secretion occurs during urea utilization, with a putative aldehyde-alcohol dehydrogenase (Blon_2241) upregulated by *B. infantis* UMA 272 and UMA299 relative complex nitrogen. Moreover, *B. infantis* UMA272 upregulated this gene during urea metabolism relative to L-cys utilization (FDR < 0.05).

### Specific physiological networks are enriched during urea utilization

A Gene Ontology (GO) enrichment analysis was performed to determine biological processes, metabolic function, or cellular components predicted to respond to urea utilization. Comparisons were performed between cells growing on urea relative to the positive control (i.e. complex nitrogen) (Table S15), between urea and L-cysteine (Table S16), and between L-cysteine and the positive control (Table S17). In the comparison between urea and complex nitrogen, all 9 GO terms that were differentially expressed were upregulated for UMA272, whereas 6/9 differential GO terms were upregulated for UMA302, and 11/16 were significantly increased for UMA299 (Fig. S13A-C and Table S15).

More specifically, UMA272 significantly increased transport overall (z = 2.36, *p* < 0.05). This includes carbohydrate transport (z = 1.63, *p* < 0.05), nickel cation transmembrane transport (z = 1.89, *p* < 0.05), and divalent inorganic cation transport (z = 1.63, *p* < 0.05). This indicates a pivot toward acquiring extracellular molecules to efficiently utilize urea and supplement physiological needs on the defined substrate. This includes nickel capture and translocation, which is essential for urease function. Glycolytic processes were increased for both *B. infantis* UMA299 (z = 0.32, *p* < 0.05) and *B. infantis* UMA302 (z = 2.53, *p* < 0.05), potentially linked to the increased demand for ATP or providing carbon backbones for amino acid biosynthesis. The DNA stress response (i.e. SOS) was significantly increased (z = 0.95, *p* < 0.05) for *B. infantis* UMA299, indicating that a signal cascade was initiated by the stress of the less-efficiently utilized urea.

The comparison between urea and L-cysteine was performed to exclude a general response to a defined and limiting nitrogen source. Consistent with the urea-complex nitrogen comparison, general transport systems were significantly increased while UMA272 metabolized urea relative to L-cysteine (z = 0.83, *p* < 0.05). In contrast, *B. infantis* UMA299 significantly decreased general transport systems and amino acid transmembrane transport urea utilization in the same comparison (z = −1.34 and z = −1.44, *p* < 0.05) (Figure S13D-E and Table S16).

### The *B.* *infantis* metabolome responds to nitrogen sources

**Figure 6.**
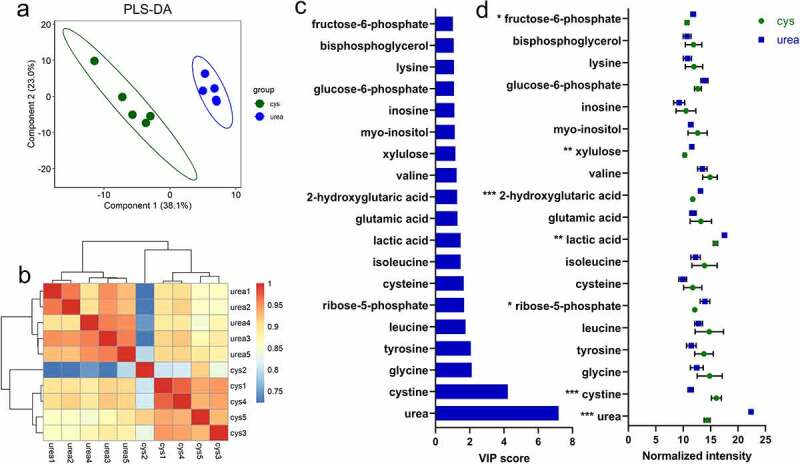
**The *B. infantis* metabolome during urea nitrogen utilization**. Partial least squares-discriminant analysis (PLS-DA) **(a)**, hierarchical clustering of metabolomes during urea and L-cysteine nitrogen utilization **(b)**, discriminatory metabolites with Variable Importance for projection (VIP) score ≥1 **(c)**, and normalized intensity of differential metabolites while *B. infantis* utilized urea as a nitrogen source (mean ± SD) **(d)**. Five biological replicates were evaluated, unpaired t-tests were performed, and *P* values adjusted by the Benjamini–Hochberg method. *, p < 0.05, **, p < 0.01, ***, p < 0.001.

Mass spectrometry-based metabolomics was used to determine specific intracellular metabolite signatures during nitrogen utilization. This non-targeted approach identified 125 metabolites across all samples. The metabolomes distinctly segregated according to nitrogen source when subjected to partial least squares discriminant analysis (PLS-DA) (Figure 6A and Fig. S14). The R^2^ (0.89 for component 1, 0.98 for component 2) and Q^2^ (0.74 for component 1, 0.91 for component 1) values calculated by leave-one-out cross-validation to confirm the PLS-DA model to ascertain differential metabolites profiles (Table S18). Significant differential metabolites were identified with a Variable Importance in Projection (VIP) score on component 1 > 1.0 combined with FDR <0.05. In total, seven metabolites were identified as significant differential metabolite between urea and L-cysteine Figure 6C-D.

Urea exhibits the highest VIP score indicating that it is a key metabolite responsible for metabolome variation. Accordingly, urea was significantly more abundant (normalized intensity) when *B. infantis* subsisted on urea compared to L-cysteine (*p* < 0.05). This indicates that urea is translocated intracellularly prior to ureolytic degradation as was hypothesized. Cystine represents the second highest VIP score responsible differentiation of urea and L-cysteine metabolome profiles. Cystine is an oxidized dimer formed from two cysteine molecules. Cystine was significantly higher when *B. infantis* subsisted on L-cysteine compared to urea, representing a metabolic signature for L-cysteine nitrogen metabolism in *B. infantis*.

Interestingly, most differentially expressed metabolites (>57.0%) between urea and cysteine were identified as carbon metabolism metabolites, including fructose-6-phosphate, xylulose, lactic acid, and ribose-5-phosphate. All four metabolites were significantly increased when *B. infantis* subsisted on urea, indicating an increase in carbon metabolism during urea utilization despite utilizing the same carbohydrate source. This is consistent with the transcriptome in that a general upregulation of carbon metabolism genes was observed during urea utilization. Moreover, ^15^N-labeled proteins involved in carbohydrate degradation were identified (Table S3). In summation, urea nitrogen metabolism is interconnected with carbon metabolism and urea utilization generally increases flux through carbon metabolic pathways compared to L-cysteine nitrogen utilization.

### Urea nitrogen utilization modulates *B.* *infantis* adhesion to Caco-2 cells

**Figure 7.**
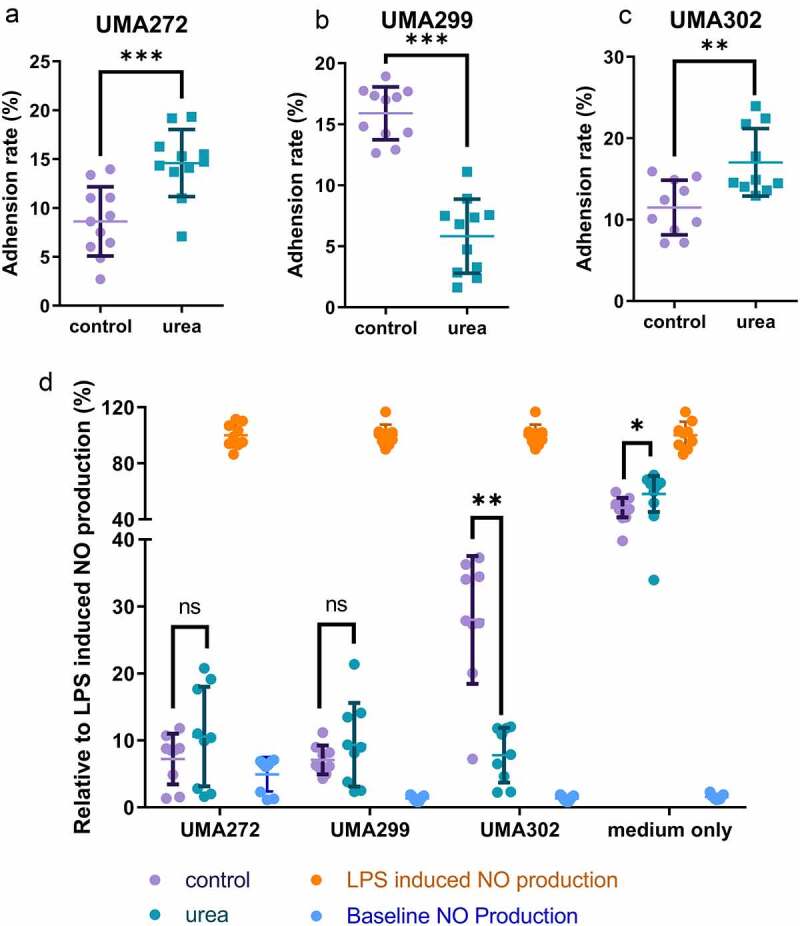
Interactions with host cells while *B. infantis* utilizes urea nitrogen. Adhesion to Caco-2 cells were assessed for *B. infantis* strains utilizing urea nitrogen including UMA272 (A), UMA299 (B), and UMA302 (C). Mitigation of LPS-induced inflammation as measurred by nitric oxide production (D). Results are presented as mean ± SD from three independent experiments (9–11 biological replicates total). Unpaired or Welch’s t-test was performed to compare urea and control conditions. *, *p* < 0.05, **, *p* < 0.01, ***, *p* < 0.001.

The interconnectedness of urea metabolism with *B. infantis* persistence in the breastfed infant gut was evaluated as a function of adhesion to epithelia-derived Caco-2 cells. More specifically, UMA272, UMA299, and UMA302 were subjected to growth on urea as a primary nitrogen source and adherence to a monolayer was determined by a previously developed qRT-PCR method (Figure 7A).^28^ Interestingly, two *B. infantis* strains increased their adherence to host cells significantly while utilizing urea (UMA272: 14.60% ± 3.43% from 8.62% ± 3.54%; UMA302: 17.04% ± 4.14% from 11.50% ± 3.36%) (*p* < 0.05). This indicates that urea metabolism initiates a response that enhances adhesion with implications for infant-microbial interactions. Conversely, *B. infantis* UMA299 significantly reduces its adherence efficiency as a result of utilizing urea nitrogen (5.83% ± 3.04% from 15.89% ± 2.16%) (*p* < 0.05). Again, this divergent phenotype is consistent with other interactions between the strain and human milk molecules.

### Urea promotes *B.* *infantis* anti-inflammatory properties in an *in* *vitro* host-microbe model

An *in vitro* cell culture model was used to investigate if urea metabolism impacts inflammatory outcomes in the host. This model leverages the contributions nitric oxide (NO) makes to the inflammatory response. Lipopolysaccharide (LPS) significantly increases NO production by 34.62-fold in RAW 264.7 cells and provides the baseline to which experimental conditions were compared (Figure 7B). Spent culture collected at stationary phase from both urea and complex nitrogen significantly decreased LPS-induced NO production in a dose-dependent manner (*p* < 0.05) (Figure 7B and Figure S15). A total of 15% spent culture media from all three strains fully mitigated LPS-induced NO production and thus inflammation (Figure 7B). This indicates that bifidobacteria exert a general anti-inflammatory effect under the conditions tested. Interestingly, *B. infantis* UMA302 utilizes urea most efficiently (Figure 1C), while also significantly decreasing inflammation. It is notable, for UMA302, lactate dehydrogenase (Blon_1090) was significantly upregulated by urea nitrogen metabolism (log2FC = 0.47, FDR <0.05). This is a key gene for indolelactic acid biosynthesis which contributes to *B. infantis* anti-inflammatory properties^29^ In aggregate, when *B. infantis* utilizes urea it produces metabolites that potentiates a stronger anti-inflammatory effect than under control conditions.

## Discussion

Scientific characterization of bifidobacterial metabolism is largely restricted to interactions with carbohydrates, and thus carbon utilization has been the central focus to date. Accordingly, *B. infantis* consumption of indigestible oligosaccharides secreted in human milk represents a key paradigm in dietary interactions with host-microbial systems early in life, and potentially throughout human development. Dietary nitrogen, conversely, remains understudied as it pertains to specific microbial populations that assemble in consortia along the GIT. Thus urea nitrogen delivered in relatively high concentrations in human milk is a potential source of nitrogen to microbiota and their infant host.

*B. infantis* strains harness exogenous urea as evidenced by their growth phenotype on this substrate. This is accomplished through a chromosomally encoded urease gene cluster which catalyzes the rate-limiting hydrolytic step to deliver ammonia to downstream anabolism. Accordingly, the broad distribution of isotopically labeled urea nitrogen throughout the proteome clearly demonstrates utilization. Inter-strain variation is evident in the transcriptional response during urea utilization, with urease-associated genes upregulated universally. Furthermore, preliminary *in vitro* impacts on host-microbial interactions suggest strain variation in adherence to cellular surfaces and mitigation of LPS-induced inflammation.

We previously documented the urease gene cluster as a conserved feature of the *B. infantis* genome which, along with the high concentration of urea in human milk, prompted the hypothesis that urea nitrogen is salvaged by *B. infantis* in the breast-fed infant gut.^6,8^ This was further informed by the analogous process described in ruminant mammals, albeit performed within a vastly divergent gastrointestinal tract.^30^ Furthermore, urease gene signatures are significantly enriched in the metagenomes of Malawian and Amerindian infants.^31^ This suggests that commensal ureases may have had a functional role in human development or, alternatively, signals importance in other geographic/genetic contexts distinct from westernization. In an independent and parallel effort, *B. infantis* ATCC17930 (i.e. the parent strain of UMA299) was demonstrated to grow on urea as a primary nitrogen source^32^ which is consistent with the findings presented herein. Interestingly, urease genes are overrepresented in the breastfed infant gut microbiome relative to formula-fed infants in an analysis of metagenomic databases.^32^ This indicates a link with microbial ureases and exogenous urea delivered early in life and moreover suggests a functional role within the infant gut environment. In addition, *B. infantis* exhibits significantly lower urease activity relative to bacterial pathogens that deploy urease as a virulence factor.^33,34^ This includes *Helicobacter pylori* which secretes ammonia extracellularly to increase local pH in their microenvironmental to promote fitness.^33^ In contrast, *B. infantis* liberates ammonia from urea to be assimilated into amino acids as evidenced by the ^15^N-labeled proteome analysis. Providing the infant with nitrogenous products derived from urea nitrogen (e.g. BCAAs) through either secretion or cell lysis represents a potential significant beneficial function within the gut.

*B. infantis* L-cysteine auxotrophy is a minor challenge in defining nitrogen utilization phenotypes. It is difficult to disentangle its utility as a nitrogen source fed into general anabolism from its value as an essential monomer in protein biosynthesis. This is further exacerbated by *in vitro* microbiological approaches that investigate large clonal populations that cannot account for metabolic heterogeneity. Although cells are designated within a crude growth cycle stage (e.g. mid-exponential), individual cells are in different stages of development and exhibit divergent phenotypes.^35^ Thus defining an appropriate limiting L-cysteine concentration is tuned against the aggregate physiology of the culture and this amino acid could supplement a diminished fraction of nitrogen demand. Nevertheless, the controlled experiments demonstrate that urea nitrogen is utilized. Most importantly, isotopically labeled urea nitrogen is incorporated into the proteome to *incisively* demonstrate this phenotype excludes other explanations for the growth. Single-cell approaches would add a measure of granularity, albeit only to further refine the phenotype in a system that possesses other inherent caveats and limitations. Thus the conclusion would remain unchanged, *B. infantis* utilizes urea nitrogen and transforms it into products usable by heterologous members of the microbiome and the infant host.

*B. infantis* utilization of urea nitrogen enables novel research and applications in infant and human nutrition in general. The initial opportunity is to reconsider human milk substitute formulations and other nutritional interventions to account for host-microbial nitrogen cycling in concert with other factors, including HMOs. This will be further informed by identifying target infant populations that could benefit by leveraging microbiota-mediated urea nitrogen recycling. This precision nutrition approach necessitates further fundamental studies on *B. infantis* nitrogen utilization phenotypes, the mechanisms deployed by heterologous microbiota participating in nitrogen cycling, as well as the interface that the emergent physiology of the microbiome presents to its infant host.

As breast milk provides adequate protein nitrogen that remains stable despite maternal nutritional status,^36,37^ additional or alternative impacts of bifidobacterial-mediated urea nitrogen salvaging are possible. This includes resolving to what degree urea produced endogenously from infant nitrogen homeostasis is salvaged. In addition, *B. infantis* might also recycle nitrogen to urea as evidenced by the increase in intracellular urea while *B. infantis* utilized the L-cystine medium compared to the complex nitrogen control. Although this remains hypothetical in the absence of canonical genes attributed to the urea cycle in other organisms. Moreover, access to modern medical and nutritional practices may diminish the role of urea nitrogen recycling in certain infant populations. In addition, urea nitrogen salvaging may benefit the human host indirectly through promoting syntrophic interactions with microbiota populations that produce quantifiable soluble products for host use or signaling. This intriguing hypothesis entails bifidobacterial nitrogen salvaging initiating a response that directs microbiome function with downstream consequences to the immune system, the gut-brain axis, and other host-microbial interactions in addition to nitrogen metabolism.

## Materials and methods

### Routine bacterial propagation and growth phenotype assay

Bifidobacteria were grown on de Mann-Rogosa-Sharp (MRS) broth supplemented with 0.05% w/v L-cysteine (Sigma-Aldrich) and incubated in a vinyl anaerobic chamber (Coy Laboratory Products) at 37°C (5% CO_2_, 5% H_2_, and 90% N_2_). Bacteria were sub-cultured twice in MRS medium prior to each assay.

To test nitrogen utilization, modified MRS medium (mMRS) was prepared with lactose (2%), dipotassium phosphate (0.2%), magnesium sulfate (0.02%), manganese sulfate (0.005%), sodium acetate (0.5%), tween-80 (0.1%), and supplemented with either urea or complex nitrogen (i.e. peptone, yeast extract, and ammonia citrate) as the positive control. All media was supplemented with 0.03–0.04% w/v L-cysteine,^38^ which is an essential amino acid that L-cysteine auxotroph *B. infantis* requires and could grow on as a nitrogen source (Fig. S16). Overnight culture was inoculated at 1% into 96-well microtiter plates and cell growth was monitored in real time by assessing optical density at 600_nm_ on a BioTek PowerWave HT plate reader every 15 min preceded by 5 seconds shaking. Three biological replicates and three technical replicates were performed per experimental condition. Bacterial growth kinetics were calculated using Wolfram Mathematica 10.3 Student Edition with the equation below.^39^ Tc is the inflection point indicating the time to reach the highest growth rate. ΔOD_asym_ is the growth level at stationary phase with k representing the growth rate.

$\Delta OD\left(t\right)\;=\;\Delta ODasym\;\;[\frac{1}{1+\exp\left[k\left(tc-t\right)\right]}-\frac{1}{1+exp\left(ktc\right)}]$

### Urease activity and ammonia concentration assays

Urease activity was assayed by the phenol-hypochlorite method.^40^ Cells pellets were harvested at exponential phase, washed 3× with chilled 25 mM pH 7.0 HEPES (Fisher), and resuspended (1 mL) prior to transfe to a lysing matrix E tube (MP Biomedicals). Bead-beating was performed on a FastPrep-24™ 5 G homogenizer (MP Biomedicals) at 3.5 m/s for 30s (3×) and chilled between rounds. Cell lysates were harvested by centrifugation (16,200 × G, 3 min, 4°C). Lysate protein concentrations were measured using Pierce BCA protein Assay kit (Thermo-Scientific) on a Nanodrop spectrophotometer (Thermo Scientific).

20 µL of lysate was incubated in 20 µL urea buffer (25 mM HEPES [pH 7.0] and 300 mM urea) at 37°C for 30 min. 75 µL of phenol with nitroprusside (Sigma-Aldrich) was added to quench the reaction. An equal volume of alkaline hypochlorite (Sigma-Aldrich) was added and mixed. After incubation at 37°C for 30 min, absorbance at 620_nm_ was measured using a plate reader (SpectraMax i3×, Molecular Devices). The concentration of ammonia liberated was calculated from a standard curve (0.0–5.0 mM). Urease activity was defined as nanomoles of ammonia/minute/milligram of protein. To mitigate the influence of ammonia released from urease-independent reactions, cell lysates were incubated in 25 mM HEPES [pH7.0] (i.e. without urea) with resultant ammonia concentrations subtracted from the experimental samples. Three biological replicates and three technical replicates were performed for each bacterial strain.

Ammonia production was assessed by a colorimetric assay kit according to the manufacturer instructions (BioVision) and performed on 50 μl supernatant collected at early stationary phase of *B. infantis* growth on the mMRS supplemented with urea or peptone (control). An ammonium chloride standard curve was used to calculate concentration on four biological replicates and two technical replicates for each strain.

### Fermentative endproduct quantification

The concentration of lactate, acetate, formate, and ethanol were determined by high-pressure liquid chromatography (HPLC) analysis. Cell-free supernatants were obtained at early stationary phase of *B. infantis* growth on the mMRS without sodium acetate supplemented with urea or peptone (control). The supernatant was filtered through a 0.22 µm ﬁlter following centrifugation and stored at −20°C prior to analysis. Analytes were quantified on a Schimadzu HPLC system equipped with a Refractive Index Detector 20A. Separation was performed on an Aminex HPX-87 H column (7.8 mm ID × 300 mm, Bio Rad Laboratories) at 30°C in 5 mM H_2_SO_4_ at a flow rate of 0.6 ml/min. Metabolite concentrations were calculated from curves generated from external standards (Sigma-Aldrich) at six concentrations (0.5, 1, 5, 10, 20, and 50 mM). Four biological replicates and two technical replicates were performed.

### Isotopically-labeled urea proteomics

*B. infantis* UMA272 was subjected to growth on dual-labeled N^15^ urea at 2% (Cambridge isotope) in mMRS supplemented with L-cysteine. Cultures were harvested at mid-exponential phase, centrifuged, and the pellet was washed 3× in PBS and stored at −80°C. The pellet wasr resuspended in 1× SDS lysis and solubilization buffer (Protifi LLC) and sonicated using Bioruptor pico (Diagenode) at high power for 10–15 min (sonication cycle: 30 sec ON, 30 sec OFF). The cell lysate was harvested by centrifugation at 13,000 g for 8 min and protein concentrations measured using the Pierce BCA protein Assay kit (Thermo-Scientific) on a Nanodrop spectrophotometer (Thermo Scientific).

The S-Trap micro universal mass spectrometry sample prep kit (Protifi) was used according to manufacturer instructions. Samples were reduced, alkylated, trypsin digested, and clean-up was performed according to the manufacturer’s instructions. Briefly, 30–40 μg protein was loaded onto the spin column, washed with wash buffer, and mixed with digestion buffer containing mass spectrometry-grade trypsin (Promega) at a 1:50 ratio (wt/wt). Samples were incubated in a water bath at 47°C for 1 hr followed by overnight incubation at 37°C. Peptides were eluted by serial additions of 50 mM TEAB, 0.2% formic acid, and 0.2% formic acid in 50% acetonitrile. Eluted peptides were pooled and evaporated in a SpeedVac to dryness and stored at−80°C until further analysis.

### Mass spectrometry analysis

Tryptic digests were purified with ZIPTIP C18 pipette tips (Merck Millipore) prior to analysis on the Easy-nLC 1000 nanoLC system coupled to a Orbitrap Fusion mass spectrometer (Thermo Scientific). Peptides were separated by a 90-minute gradient of increasing acetonitrile concentration and injected at a rate of 300 nl/min.MS1 data was acquired at 60,000 resolution in the orbitrap and MS/MS data was acquired by selecting the 5 most intense ions for fragmentation, using a nominal CID collision energy of 35%. Data were processed using Proteome Discoverer 2.0 (Thermo Scientific). MS/MS data was queried against a database generated from the uniprot ATCC15697 accession number (https://www.uniprot.org/proteomes/UP000001360, Proteome ID: UP000001360). Peptide and protein identifications were validated with Scaffold (version 4.8.5, Proteome Software Inc.). A -log e score above 2.0 was deemed acceptable for peptide identification by X! Tandem, with greater than 99.0% probability with at least 2 identified peptides was the threshold for protein identification. Peptide probabilities from X! Tandem and Sequest were assigned by the Peptide Prophet algorithm (1, 2). Proteins that contained similar peptides and could not be differentiated based on MS/MS analysis alone were grouped to satisfy the principle of parsimony.

Proteins were annotated with Gene Ontology terms using UniProt ID mapping. KEGG metabolic pathway were reconstructed using KO identifiers for each protein mapped on KEGG Mapper. MS/MS data was further analyzed using Peaks studio software (Version 10.5, Bioinformatics solutions Inc.) against ATCC15697 allowing for up to 3 missed cleavages. A mass tolerance of 15 ppm was allowed for precursor ion and 0.5 Da for-fragment ions with peptide hit threshold of ≥20 (−10logP).

A peptide identification filter of FDR 1.0% was set for protein identification based on at least 1 unique peptide for qualitative analysis using the Peaks software package. Cysteine carbamidomethylation was specified as a fixed modification and methionine oxidation and ^15^*N*-labeling as variable modification. Proteins detected in at least two of the biological replicates with high scores was included in the analysis.

### RNA-seq transcriptomics

*mRNA extraction and purification*

Bifidobacteria were harvested at mid-exponential phase and biomass was immediately pelleted at 12,000 g for 1 min, resuspended in 1 ml of RNAlater, and incubated at 4°C overnight and stored at −80°C. Cells were pelleted at 10,000 g for 2 min and washed twice with PBS to remove RNAlater. The Ambion RNAqeous kit (Thermo Fisher Scientific) was used to extract total RNA following manufacturer instructions with the addition of a bead-beating step (FastPrep-24TM 5 G MP Biomedicals Inc). Total RNA was immediately subjected to DNase treatment using 1 μl of DNase I for 30 min. RNA integrity was checked by the High Sensitivity RNA analysis ScreenTape Assay on a Tape Station 2200 (Agilent Technologies). Samples with an RNA integrity number (RIN) above 7 were selected for further processing. Genomic DNA contamination was assessed by quantitative real-time PCR (qRT-PCR) with universal bacterial 16S primers, Uni334F and Uni514R.

*Library construction and sequencing*

Ribosomal RNA was removed using the Ribo-Zero rRNA Removal kit (Illumina) and purified with the RNeasy Minelute Cleanup kit (Qiagen). rRNA depletion efficacy was determined with the High Sensitivity RNA analysis ScreenTape Assay (Agilent Technologies). Whole transcriptome libraries were constructed using TruSeq Stranded mRNA Library Preparation Kit (Illumina) following manufacturer’s instructions. Barcoded libraries were quantified by Qubit dsDNA BR Assay (Life technology). Library quality was verified by the DNA analysis ScreenTape Assay (Agilent Technologies). Libraries were pooled in equimolar concentration, diluted to 4 nM, and denatured immediately prior to sequencing on an Illumina NextSeq (NextSeq 500/550 High Output v2 kit (150 cycles); paired-end sequencing; 20% PhiX spike-in).

### Bifidobacterial transcriptome analysis

#### Gene feature counting

The quality of total reads sequenced were assessed by FastQC^41^ and aligned to *Bifidobacterium longum* subsp. *infantis* ATCC15697 (NC_011593.1) using bowtie2/2–2.1.0.^42^ The resulting SAM files were converted to BAM files and sorted through SAMtools 1.2.^43^ Read counts corresponding to annotated genes was performed using HTSeq 0.6.1.^44^

### Differential gene expression

Differentially expressed genes were identified using the R package (3.3.1) DESeq2^45^ from the HTSeq output. Whole transcriptome comparisons were performed by hierarchical clustering using Euclidean distance within DESeq2 and visualized by pheatmap.^46^ Principal component analysis and volcano plots were created and visualized by PAST 3.1.^47^ Gene expression was calculated from regularized log transformation of counts and visualized in a heatmap by MeV 4.9.0 (TM4, Boston, MA, US).^48^ Benjamini-Hochberg adjusted p-value (FDR) <0.05 differences were considered statistically significant.

*Functional annotation and enrichment analysis*

*B. infantis* ATCC15697 (NC_011593.1) was queried by BlastX (version 2.2.28) along with Actinobacteria protein-coding sequences retrieved from UniProt. The resulting BlastX hits were used for functional annotations with Blast2GO (version 3.0) with default settings.^49^ GO enrichment analysis was performed by the R package goseq.^50^ Differences with p-values <0.05 were considered statistically significant. ggplot2 was used to visualize data in R. The degree of upregulation or downregulation of a GO category or pathway was reported as a z-score according to the following function.^51^$z-score=\frac{\left(upregulatedgenecounts-downregulatedgenecounts\right)}{\sqrt{totalgenecounts}}$.

### Cell culture model of host-microbial interactions

#### Culturing conditions

Caco-2 cells (ATCC HTB37, passaged 24–33) and RAW 264.7 cells (passaged 8–15) were routinely cultured in high glucose Dulbecco’s modified Eagle medium (DMEM) (Corning) supplemented with NaHCO_3_ (Sigma-Aldrich), 1% non-essential amino acids (Gibco), 100 U/ml penicillin-streptomycin (Gibco), 10% (v/v) fetal calf serum (VWR) and 7 mM HEPES (Gibco) at 37°C in a humidified atmosphere of 5% (v/v) CO_2_ in air. Caco-2 cells and RAW 264.7 were routinely grown in 20-cm petri plates and sub-cultured at 80% confluence. The cell culture medium was changed 2–3 d as needed.

### Bifidobacterial adhesion to Caco-2 cells

Caco-2 cells were seeded in a 24-well plate at a concentration of 1.0–2.0 × 10^5^ cells/cm^2^ and maintained for 14–15 d prior to the adhesion assay. Cell monolayers were washed twice with PBS before bacterial cells were added. *B. infantis* strains were collected in exponential phase by centrifugation at 4000 g for 5 min, washed 2× with PBS and re-suspended at OD_600 nm_ = 0.5 with DMEM (without antibiotics). 0.5 ml of bifidobacteria suspended in DMEM were added and incubated at 37°C in 5% CO_2_ atmosphere for 2 hours. After incubation, monolayer cells were gently washed 2× with PBS to release unbound cells. Trypsin/EDTA was used to release cells which were collected and stored at −20°C. Adhesion was measured by qRT-PCR using primers Bif_F: CGCGTCYGGTGTGAAAG and Bif_R: CCCCACATCCAGCATCCA.^52^ Strains were tested in at least triplicate in three independent experiments.

### Caco-2 cell inflammatory status

Nitric oxide (NO) production was measured as a marker of inflammation. Accordingly, Caco-2 cells were plated in 96-well plates (1 × 10^5^ cells/well) and pre-incubated for 24 hours. Lipopolysaccharide (LPS; 1 μg/mL, Sigma-Aldrich) and experimental conditions of 1%, 5%, 10%, or 15% (v/v) bifidobacterial supernatant growing on 2% complex nitrogen or urea were incubated for an additional 24 hours. LPS treatment alone served as the negative control.

The NO concentration was measured by Griess reagent (1:1 mixture (v/v) of 2% sulfanilamide and 0.2% naphthylethylenediamine dihydrochloride in 10% H_3_PO_4_). The medium (100 μL) and Griess reagent (100 μL) were mixed and incubated for 10 min at RT. Optical density was measured at 540_nm_ and sodium nitrite (Sigma-Aldrich) was used to generate a standard curve. The MTT (3-(4,5-Dimethylthiazol-2-yl)-2,5- Diphenyltetrazolium Bromide) assay was used to determine cell viability and the NO concentration was adjusted accordingly. Briefly, MTT solution (0.5 mg/mL) was added and incubated for 2 hours. Formazan crystals were dissolved with 100 μL dimenthyl sulfoxide (DMSO), and absorbance was measured at OD = 570_nm_. All strains were tested in triplicate in three independent experiments.

## GC-TOF-MS metabolomics

Cultures (5 ml) were harvested at mid-exponential phase and quenched by 15 ml −40°C 60% (v/v) aqueous methanol. Cells were maintained at −20°C for 30 min and centrifuged for 5 min at −9°C and washed with 15 ml 60% methanol and stored at −80°C Metabolome analysis was performed at NIH West Coast Metabolomics Center. In brief, the metabolomic profiling was performed on a gas chromatograph time of flight mass spectrometer (GC-TOF-MS) platform with an auto-injector system (Gerstel). Rtx-5Sil MS column (Restek corporation) was used as the separation column for the GC (Agilent 6890). Helium was used as the mobile phase with the flow rate set at 1 mL/min. The oven temperature was held constant at 50°C for 1 min, and then ramped at 20°C/min to 330C, and held constant for 5 min. The transfer line temperature between GC and TOF-MS (Leco Pegasus IV) was set to 230°C. The TOF-MS was used with unit mass resolution at 17 spectra/sec from 80 to 500 Da at −70 V ionization energy and 1800 V detector voltage with a 250°C ion source.

Raw data files were processed by ChromaTOF (v2.32, LECO Corporation) after data acquisition without smoothing, 3 sec peak width, baseline subtraction just above the noise level, and automatic mass spectral deconvolution and peak detection at signal/noise levels of 5:1 throughout the chromatogram. The resulting absolute spectra intensities were further processed by a filtering algorithm implemented in the metabolomics BinBase database. Intensity of each metabolite was then normalized and imported into MetaboAnalyst 4.0 for further statistical analysis.^53^ The intensity of each metabolite was normalized by sum and generalized logarithm transformed. The very importance for projection (VIP) score obtained by the partial least squares-discriminant analysis (PLS-DA) combined with FDR value (adjusted *p* value) obtained from ANOVA with post hoc Tukey were used to screen significant differential metabolite.

## General statistical analyses

Bacterial growth, urease activity, ammonia production, carbon metabolites, adhesion rates, and nitric acid concentrations are presented as means (± standard deviation) and two-way ANOVA for parametric data and Welch’s t-test and Mann Whitney test for non-parametric data followed by Benjamini and Hochberg or Fisher’s LSD post hoc analysis. *p* < 0.05 was designated as statistically significant.

## Supplementary Material

Supplemental Material

## Data Availability

The RNA-seq data has been deposited in the NCBI GEO under accession number GSE155078. The mass spectrometry proteomics data have been deposited in the EMBL-EBI PRoteomics IDEntifcations Database (PRIDE) under accession number P×D019401. The GSE155078 is available at https://www.ncbi.nlm.nih.gov/geo/query/acc.cgi?acc=GSE155078. The P×D019401 is available at http://www.ebi.ac.uk/pride/archive/projects/PXD019401.
