# Supplementary material for: Bifidobacterium longum subsp. infantis utilizes human milk urea to recycle nitrogen within the infant gut microbiome
Source: Gut Microbes. 2023 Mar 26;15(1):2192546. doi: 10.1080/19490976.2023.2192546 (PMC10054289; doi:10.1080/19490976.2023.2192546)
Supplement: Supplemental Material [file KGMI_A_2192546_SM3268.zip › si figures urea sela.pdf]

Fig. S1

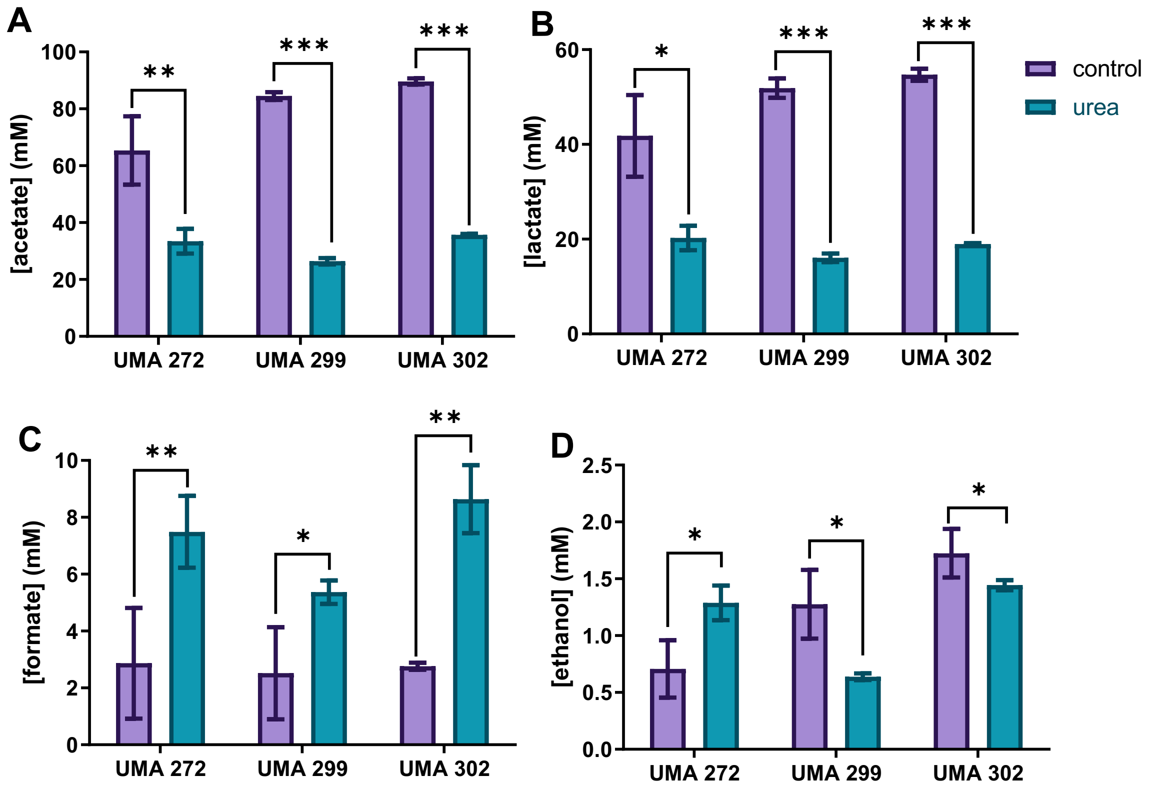

Figure S1. Fermentative endproducts secreted while utilizing urea. acetate (A), lactate (B), formate (C) and ethanol (D) production in the control and urea medium were quantified by HPLC. 4 biological replicates and technical duplicates were measured with Welch's t-test was performed. *, p<0.05. **, p<0.01, **, p<0.001

Fig. S2

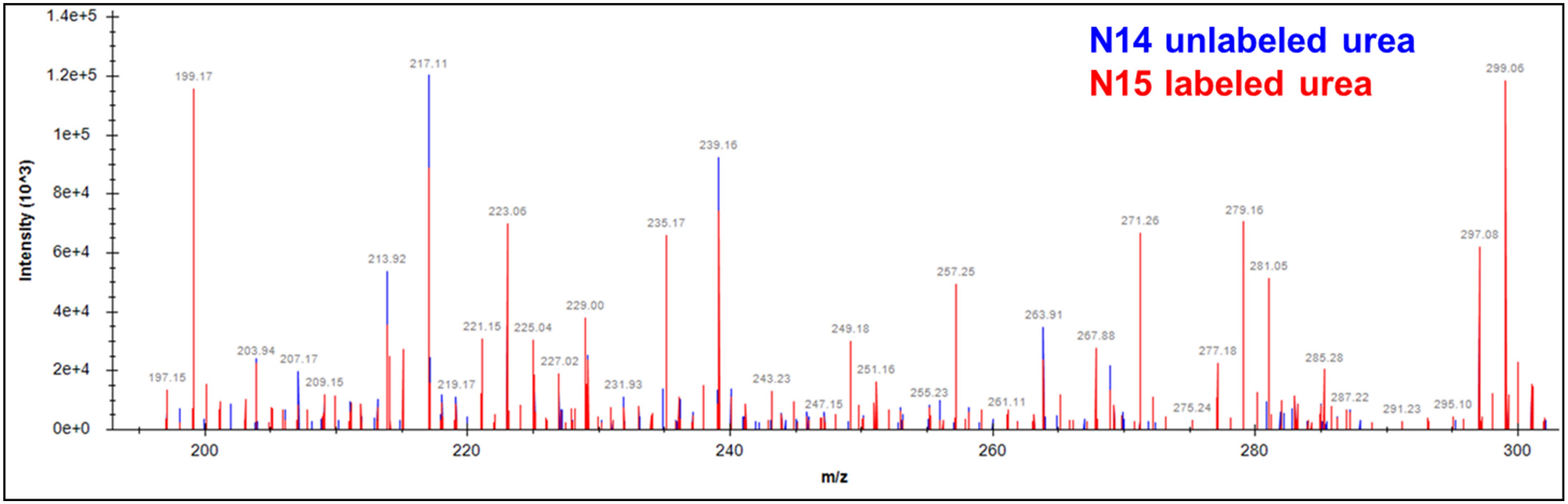

Figure S2. Mass spectrum generated from *B. infantis* UMA 272 utilizing unlabeled ^{14}N (blue) and ^{15}N labelled (red) urea. Mass to charge ratio (m/z) and intensity values are display on the x and y-axis respectively.

Fig. S3

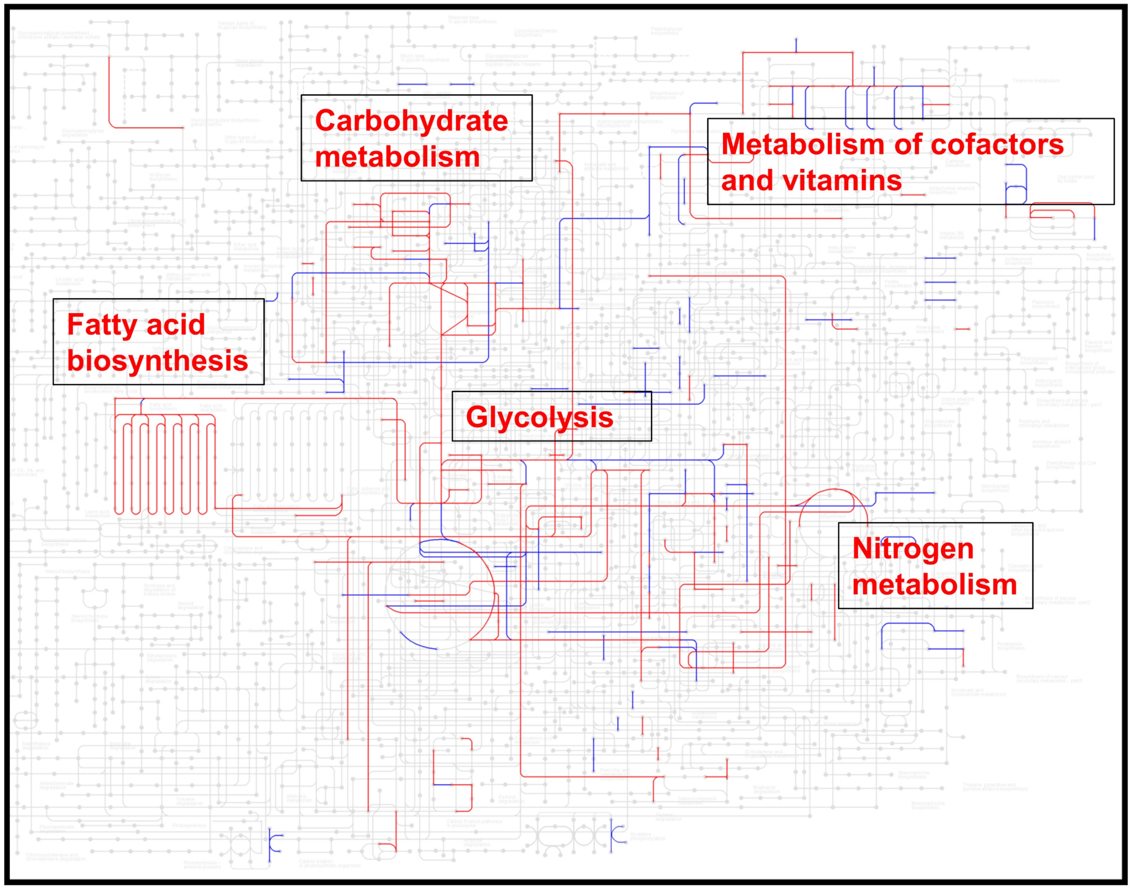

Figure S3. KEGG metabolic network map of the *B. infantis* UMA 272 proteome while utilizing labeled urea. Proteins identified in both ^{15}N labeled urea and ^{14}N urea datasets are marked in red. Proteins identified only in ^{14}N urea are displayed in blue.

Fig. S4

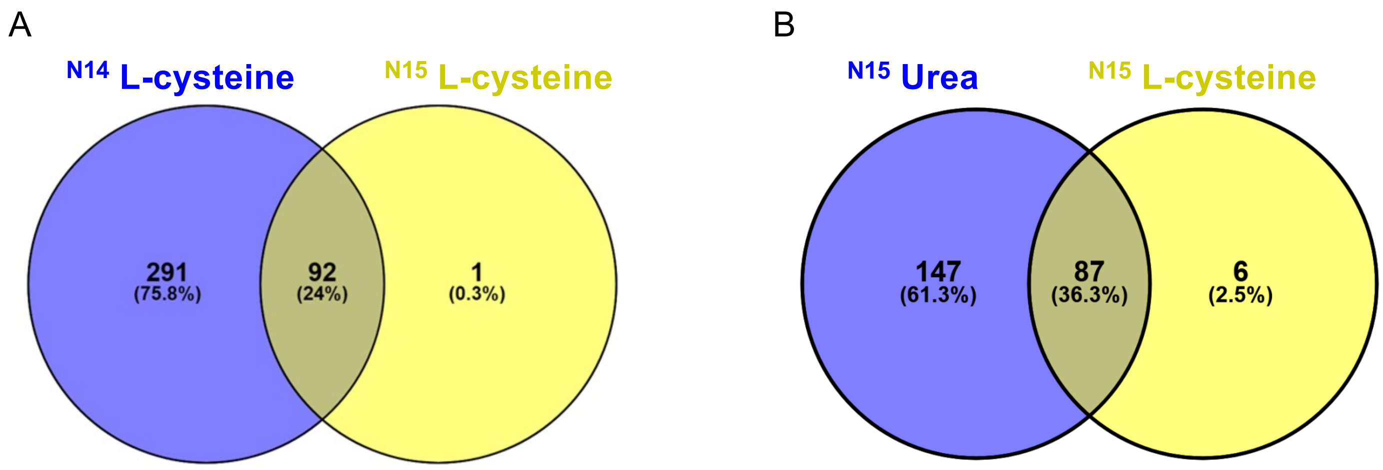

Fig. S4 Venn diagram for number of proteins identified in ^{14}N and ^{15}N labeled L-cysteine proteome. A) Approximately 24% proteins were ^{15}N labeled and were shared among both the datasets. Only ^{15}N labeled proteins were included in the labeled L-cysteine proteome analysis. These proteins were present in both labeled and unlabeled samples. B) Comparison of ^{15}N labeled urea and ^{15}N labeled L-cysteine proteins. ^{15}N labeled urea proteome contained ~97.6% of the labeled proteins compared to 36.3% of the L-cysteine proteome.

Fig. S5

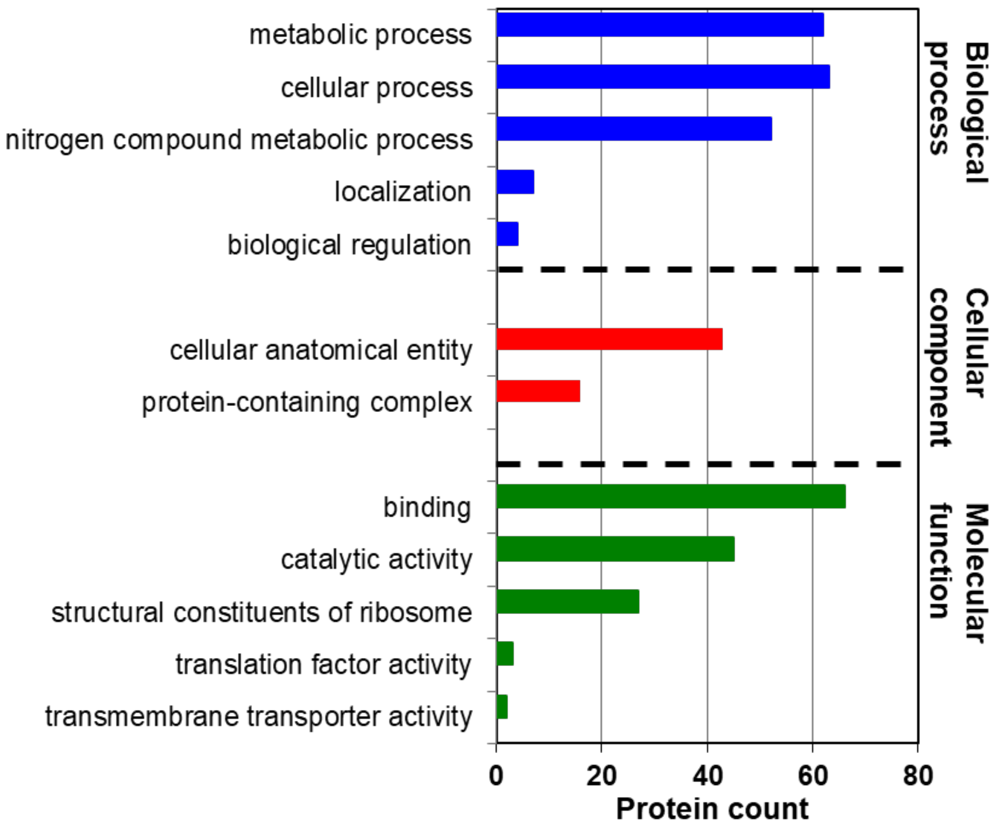

Figure S5 Gene ontology categories identified in the *B. infantis* UMA 272 proteome during ^{15}N labeled L- cysteine utilization. ^{15}N labeled protein counts are binned into specific functional categories.

Fig. S6

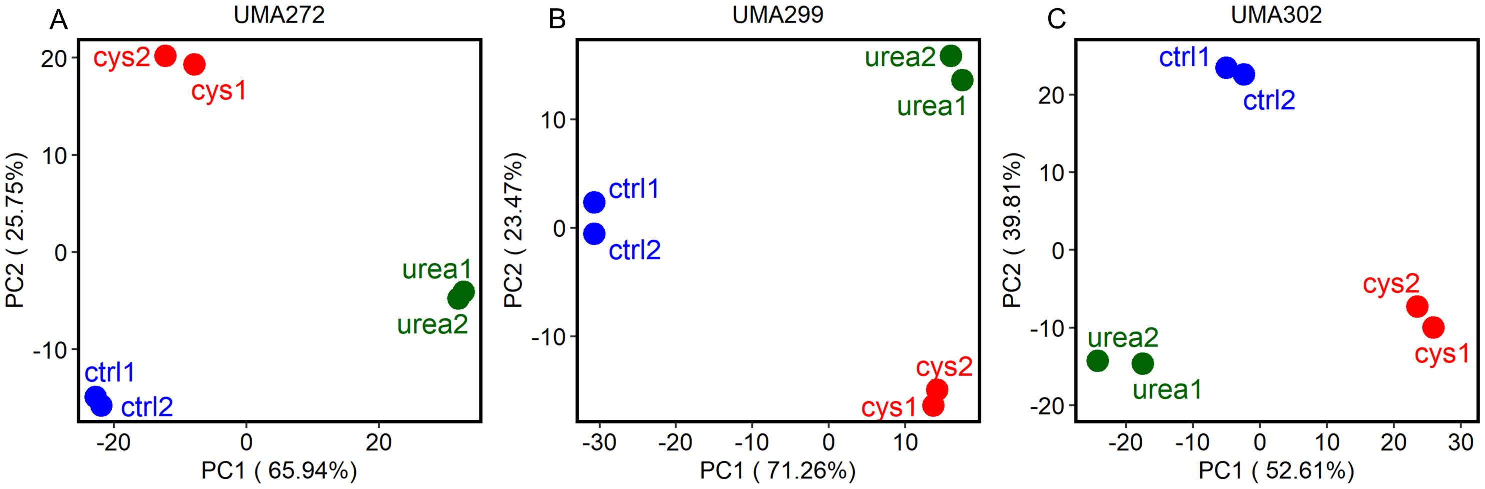

Figure S6. Principal component analysis plot of whole transcriptome response to nitrogen substrates. ctrl, complex nitrogen (control); cys, L-cysteine; 1, biological replicate one; 2, biological replicate two.

Fig. S7

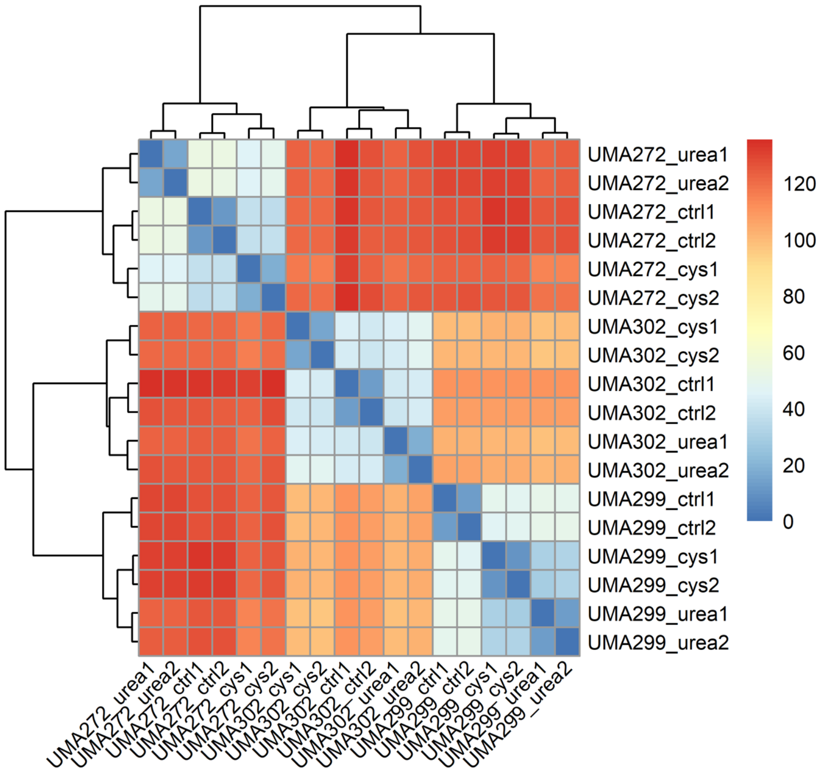

Figure S7. Whole transcriptome responses during nitrogen by *B. infantis* strains. The heatmap depicts Euclidian distances of the regularized log transformation of counts distances between transcriptomes in response to nitrogen source. ctrl, complex nitrogen (control); cys, L-cysteine; 1, biological replicate one; 2, biological replicate two.

Fig. S8

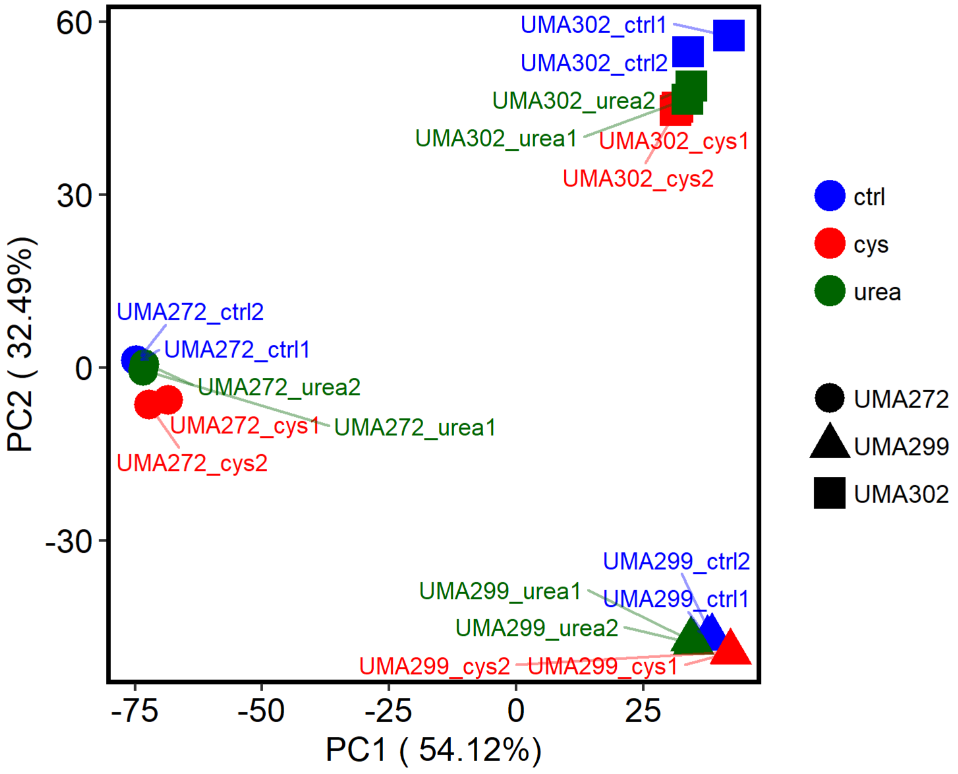

Figure S8. Principal component analysis plot of whole transcriptome responses to nitrogen substrates. ctrl, complex nitrogen (control); cys, L-cysteine; 1, biological replicate one; 2, biological replicate two.

Fig. S9

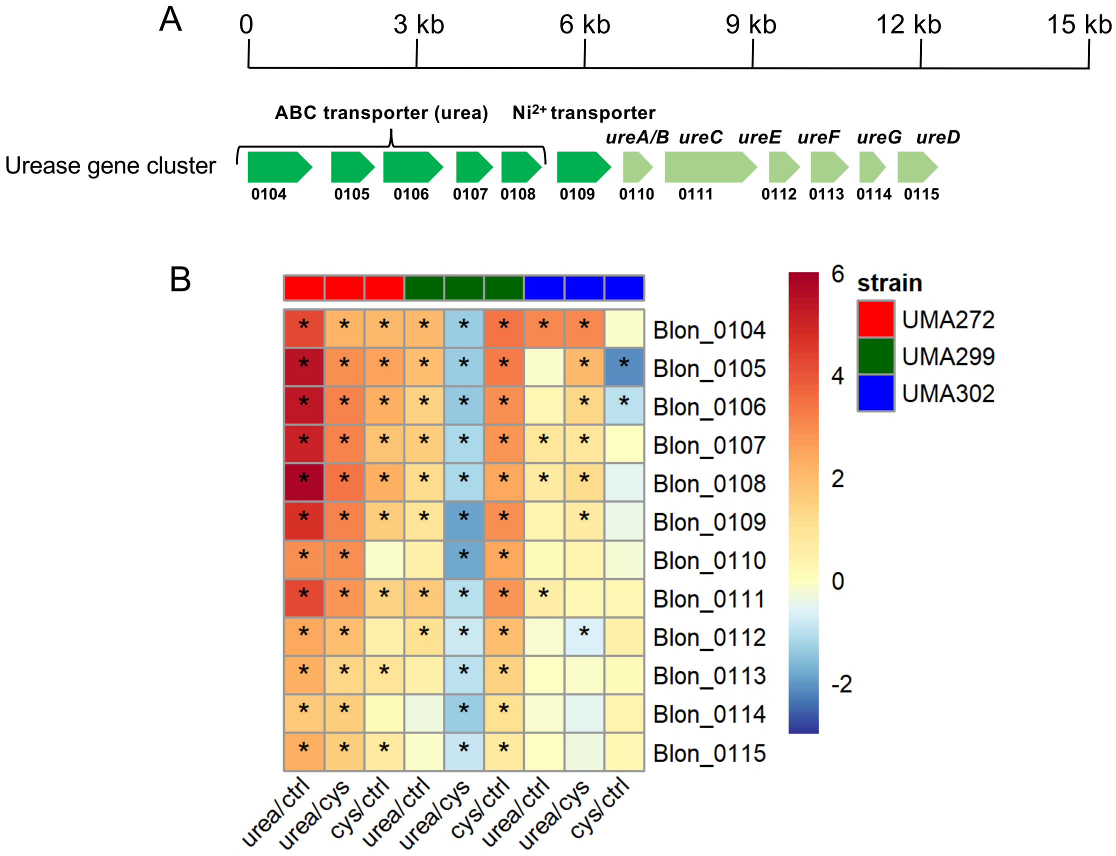

Figure S9. ***B. infantis*** **urease gene cluster (A) and urease gene cluster expression (B).** The heatmap displays gene expression for loci in the urease gene cluster based on regularized log transformation of counts calculated from R package DESeq2. cys, L-cysteine; ctrl, control.

Fig. S10

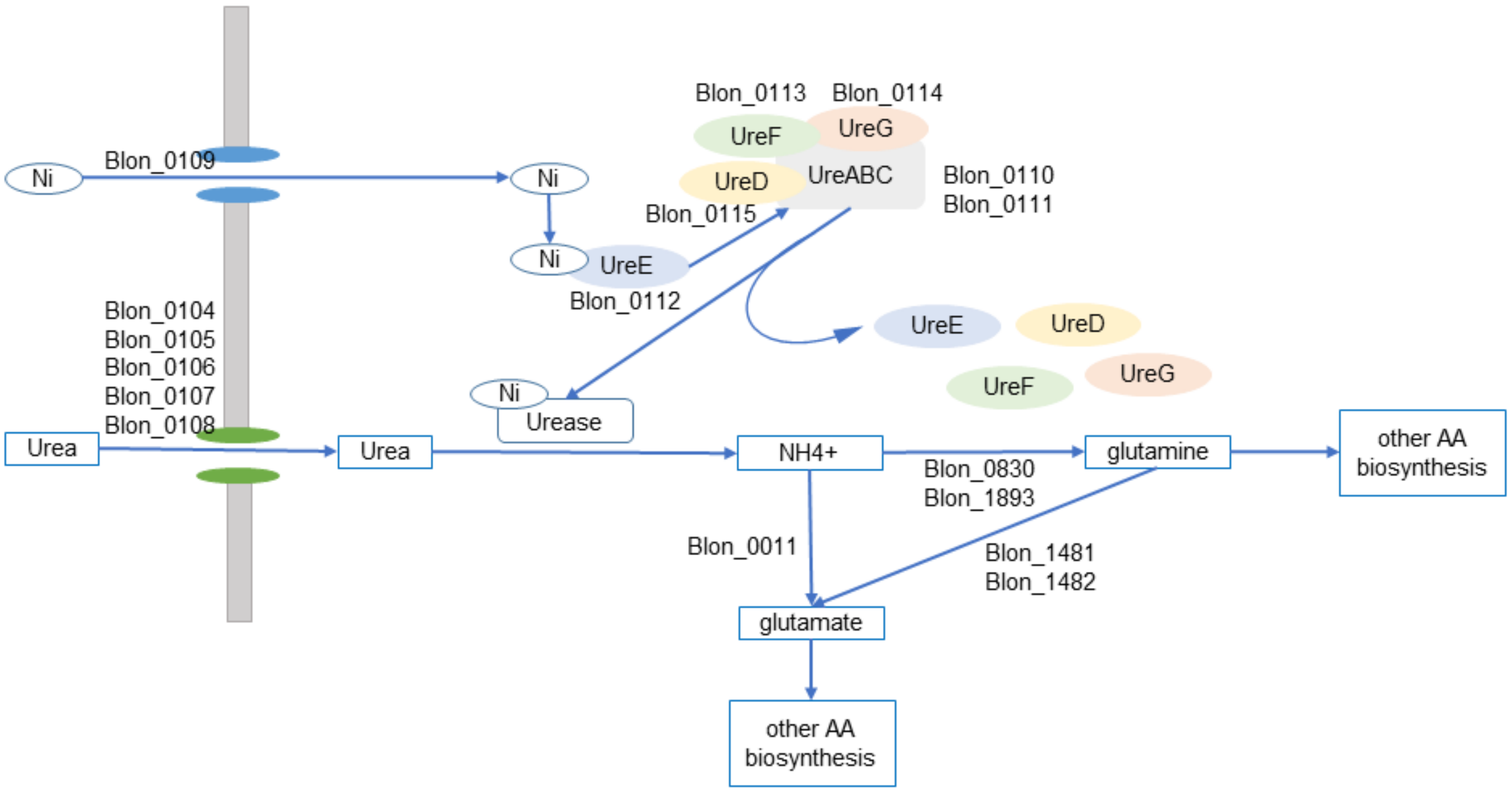

Figure S10. Putative *B. infantis* urea utilization pathway. The pathway is scaffolded from the KEGG database (http://www.genome.jp/dbget-bin/www_bget?gn:T00794). Urea transporter expression is induced by urea for intracellular translocation which upregulates urease expression.

Fig. S11

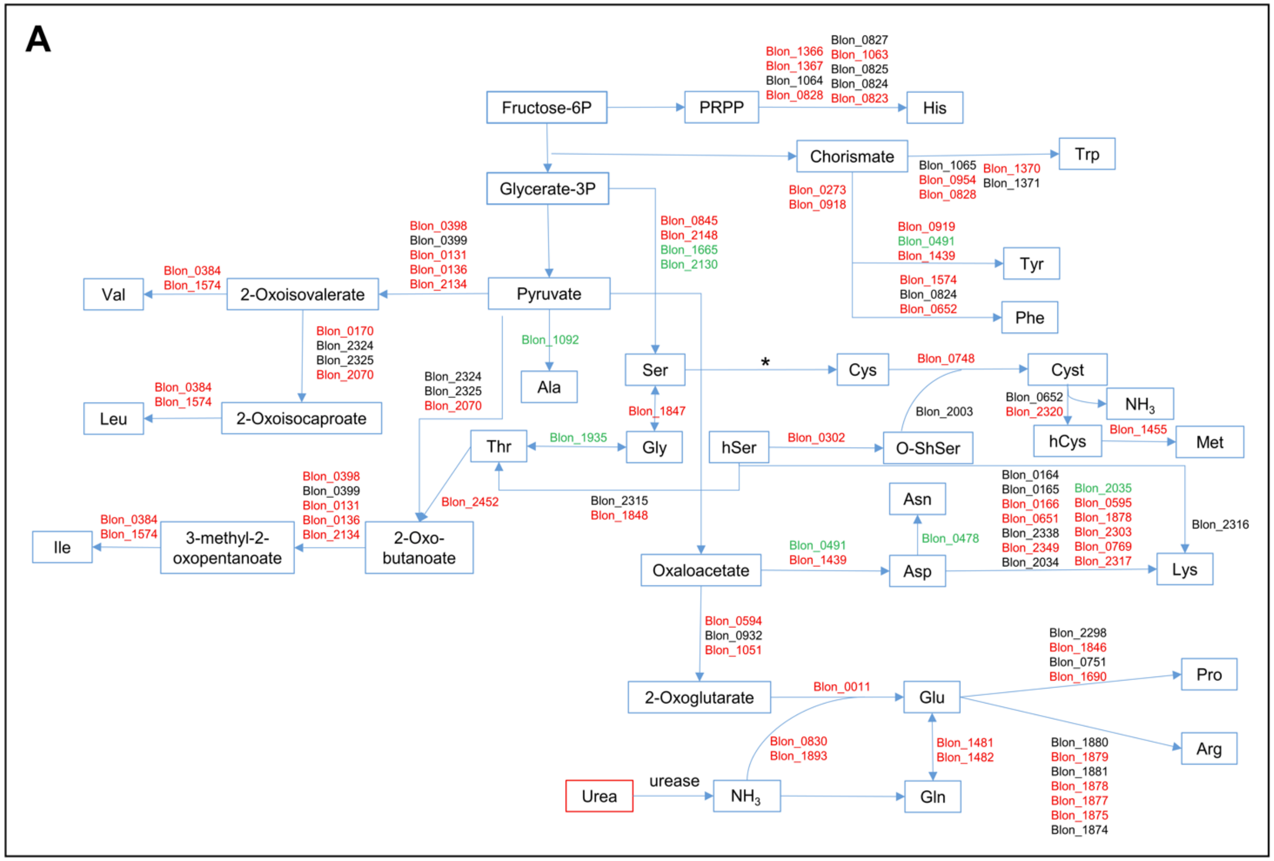

Figure S11. Amino acid biosynthetic pathways respond to urea utilization relative to the control (A) and L-cysteine (B). Genes labeled in red indicate significant up-regulation and green indicates significant down-regulation. The amino acid biosynthetic pathways are scaffolded from the KEGG database (http://www.genome.jp/dbget-bin/www_bget?gn:T00794). Genes and encoded enzymes are listed in Table S4. * conserved genes are not identified from homology

Fig. S11

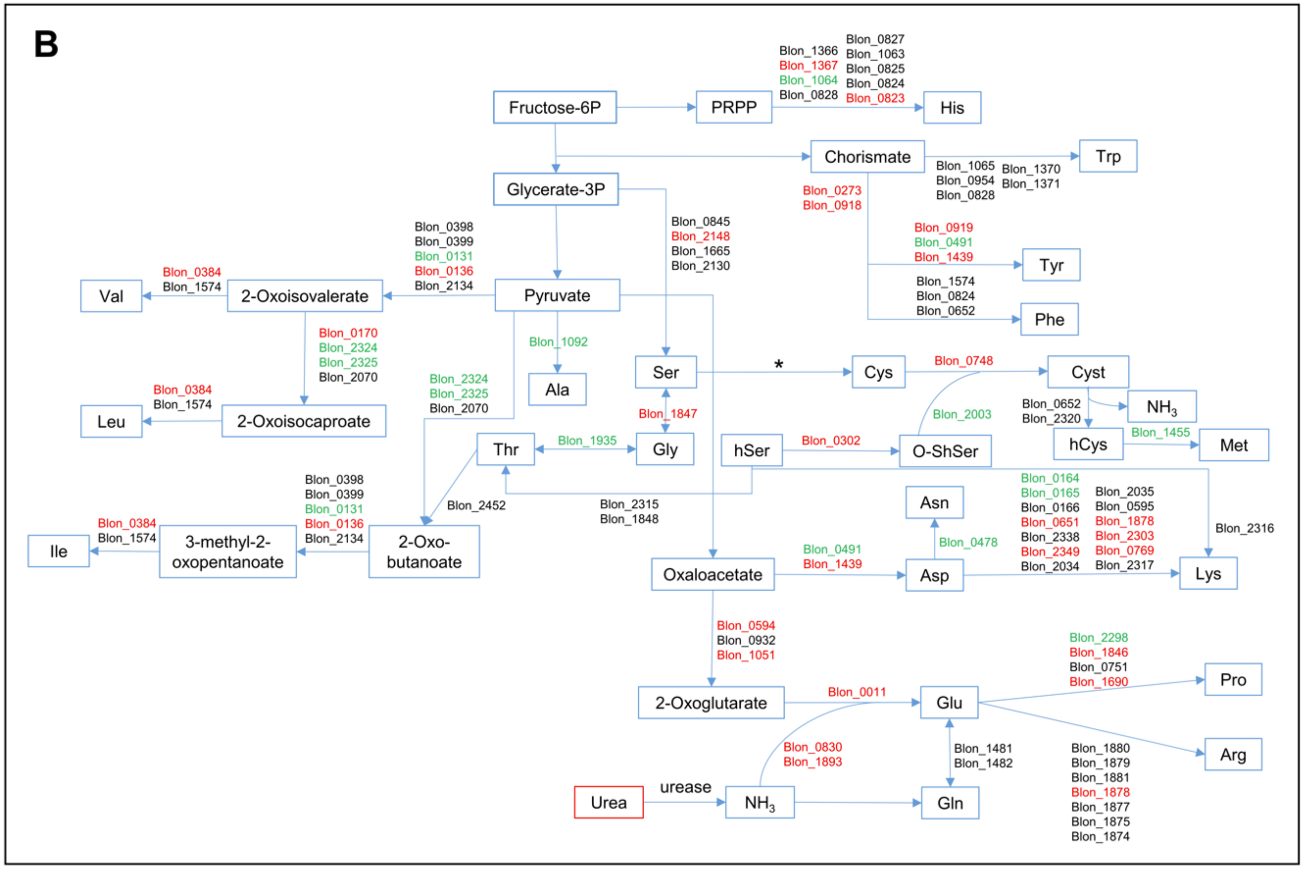

Figure S11. Amino acid biosynthetic pathways respond to urea utilization relative to the control (A) and L-cysteine (B). Genes labeled in red indicate significant up-regulation and green indicates significant down-regulation. The amino acid biosynthetic pathways are scaffolded from the KEGG database (http://www.genome.jp/dbget-bin/www_bget?gn:T00794). Genes and encoded enzymes are listed in Table S4. * conserved genes are not identified from homology

Fig. S12

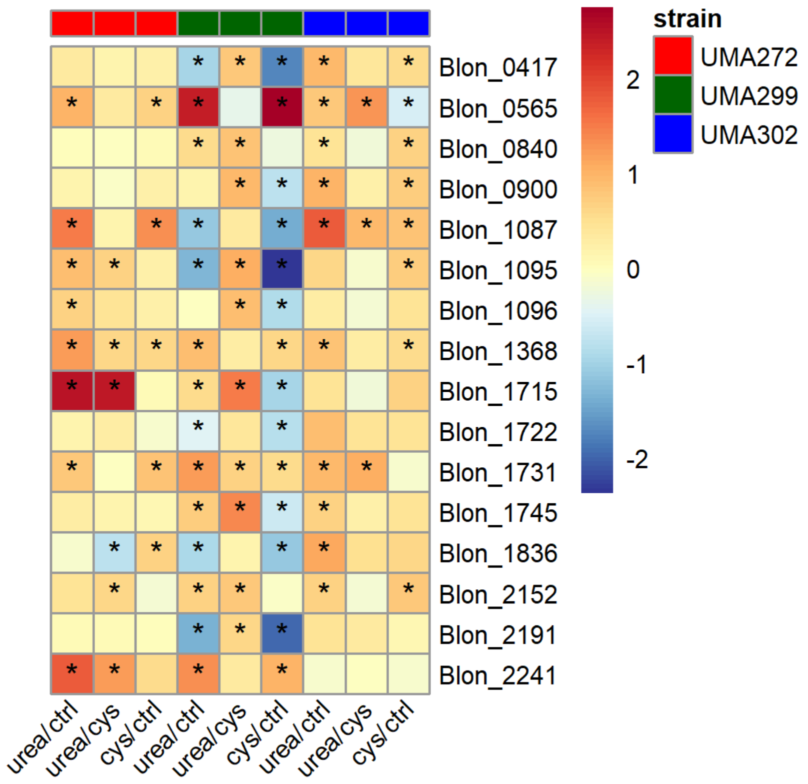

Figure S12. Gene expression of carbon catabolic genes in response to nitrogen substrates. cys, L-cysteine; ctrl, control; 1, biological replicate one, 2, biological replicate two.

Fig. S13

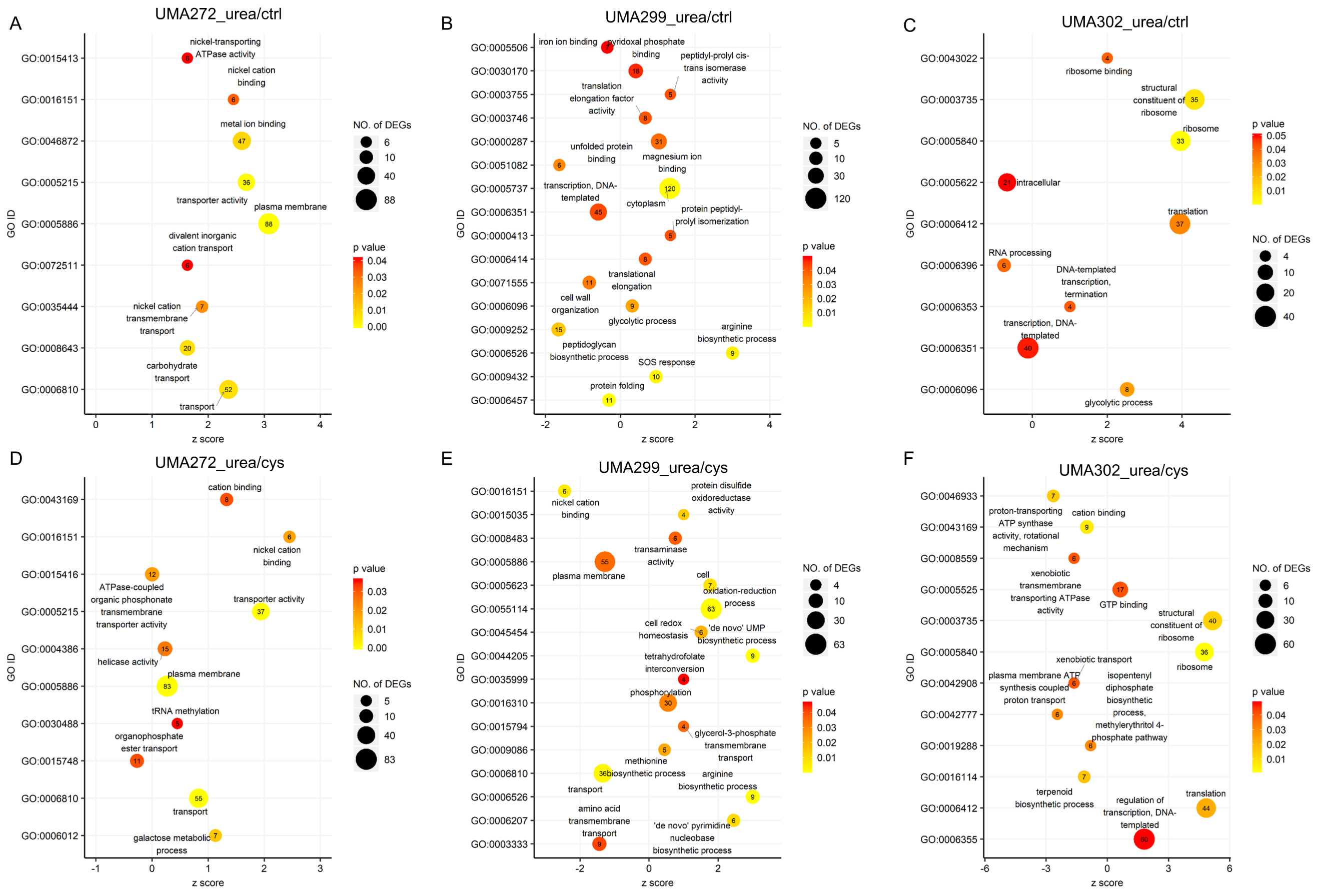

Figure S13. Gene Ontology (GO) enrichment analysis of pairwise comparisons. GO term differential expression is reported by the z-score for UMA 272 (A, D), UMA 299 (B, E) and UMA 302 (C, F). Higher z-scores indicate higher proportion of up-regulated genes by urea relative to the control (A-C) or L-cysteine (D-F). The value in each bubble indicates differentially expressed genes within the GO category.

Fig. S14

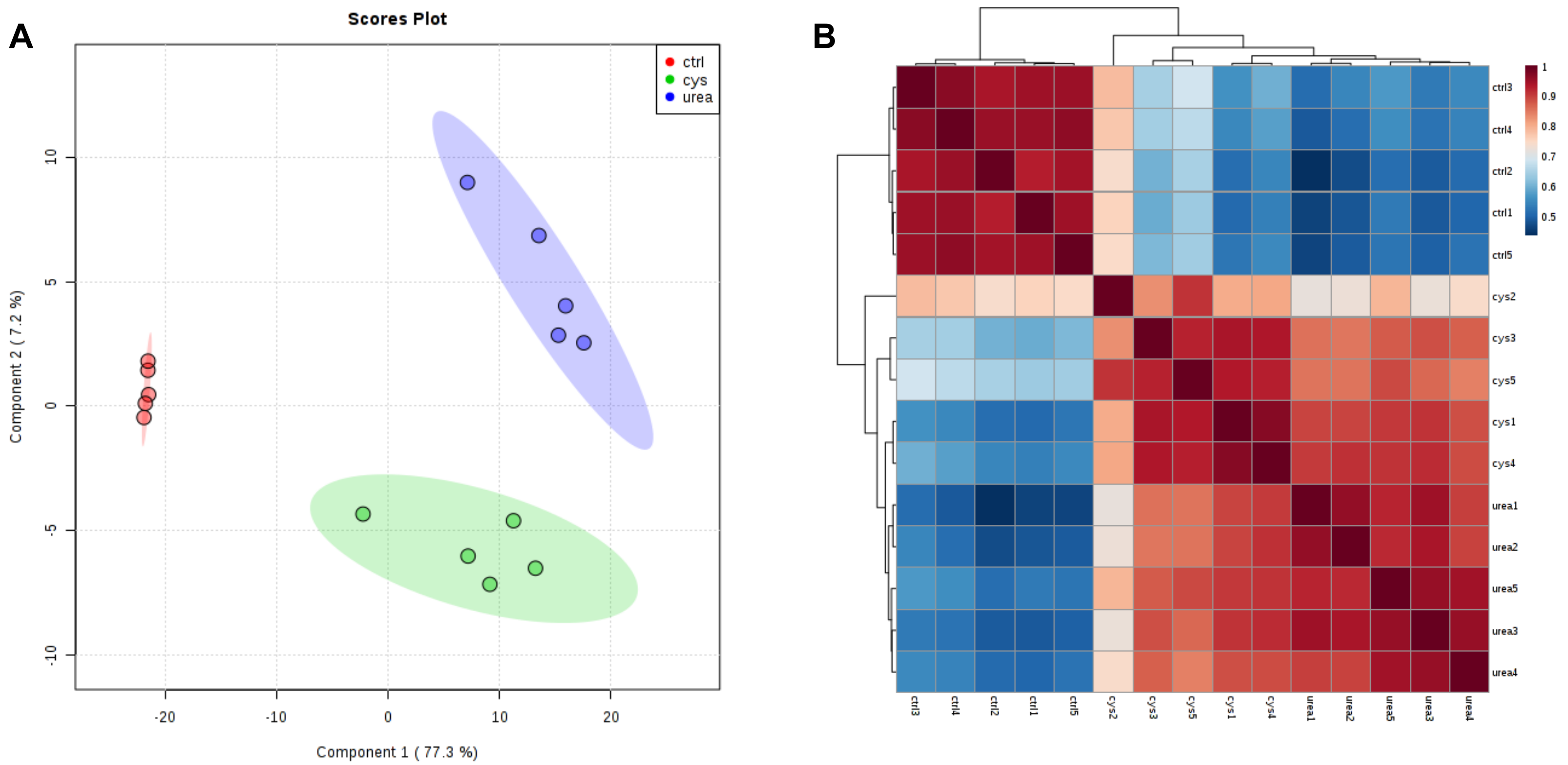

Figure S14. The *B. infantis* metabolome in response to nitrogen substrates. (A) Partial least squares—discriminant analysis and (B) clustering analysis of metabolomes exhibit a clear segregation of the metabolite profiles in response to complex nitrogen, urea, and L-cysteine.

Fig. S15

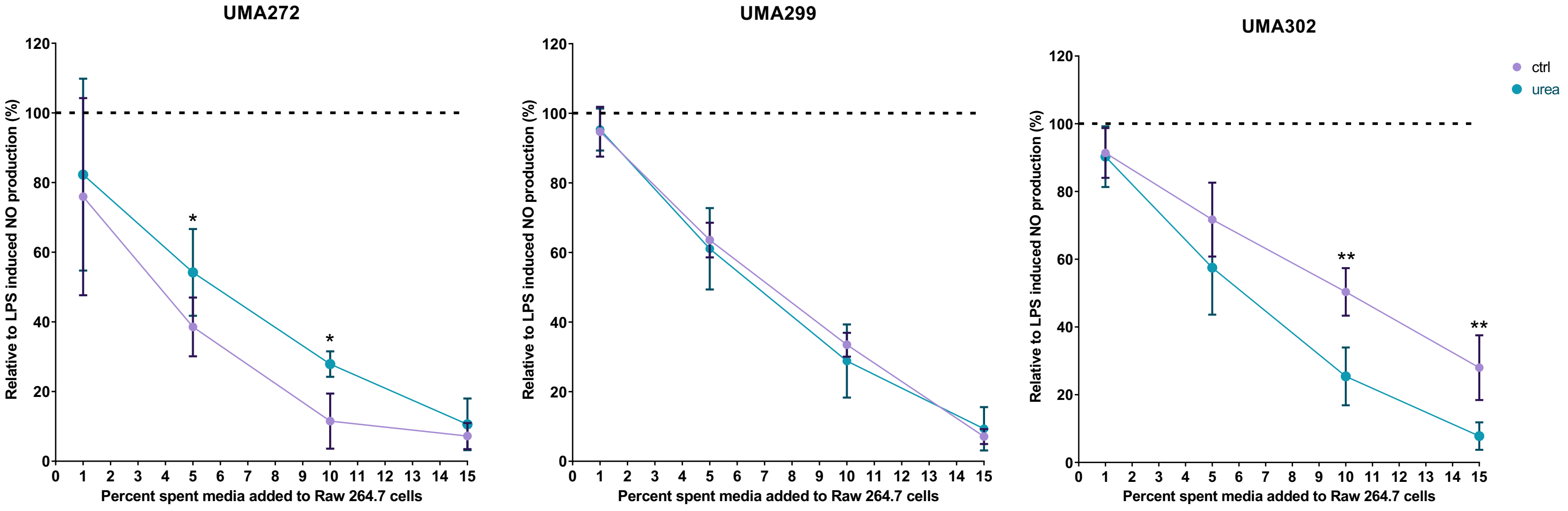

Figure S15. Dose-dependent inhibition of LPS-induced NO by *B. infantis* culture supernatants. *, p<0.05; **, p<0.01 for the comparison between urea and control spent culture.

Fig. S16

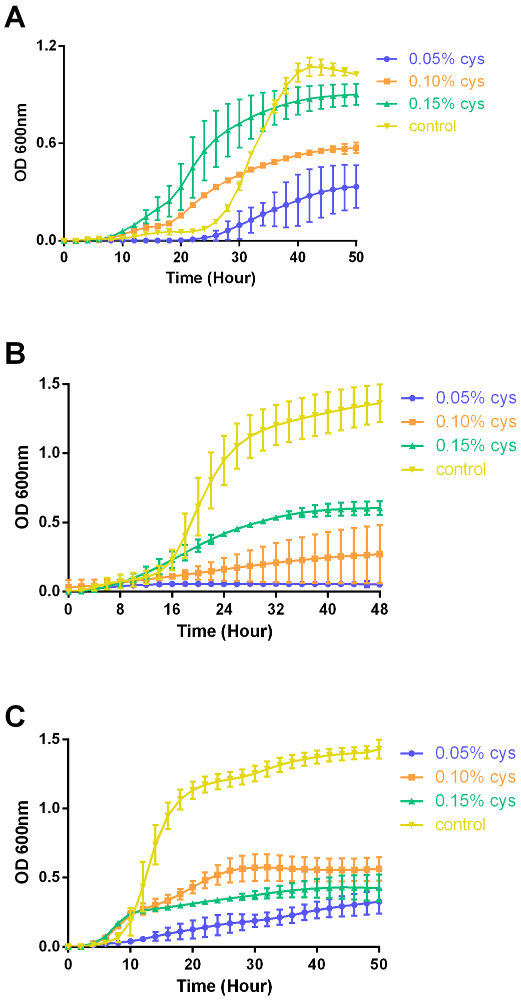

Figure S16. *B. infantis* growth on L-cysteine as the sole nitrogen source. (A) *B. infantis* UMA272, (B) *B. infantis* UMA299 and, (C) *B. infantis* UMA302 growth over time. cys, L-cysteine. 3 biological replicates and 3 technical replicates were performed. Results are presented as mean ± SD.
